# A XIV-a Conferință Națională de Balneologie și Recuperare Medicală 07 – 09 Aprilie 2017 U.M.F. „Carol Davila” - Aula Facultății de Farmacie, București

**Published:** 2017-04-07

În secolul XVIII sunt descrise pentru prima dată emanaţiile naturale de gaze cu efecte terapeutice – mofetele din Transilvania, semnalate în lucrarea „Despre vrăjitorie şi emanaţiile calde în Dacia” de medicul sibian Samuel Köleséri (1663-1732). Mofetele din Harghita au fost descrise în 1726 de italianul Luigi Ferdinando Marsigli în „Danubius Pannonica Mysicus” la Amsterdam, iar solfatarea de la Toria a fost descrisă ştiinţific în 1744 de Mátyas Bél, iar efectele terapeutice în 1761 de Ivan Fridvaldszky în lucrarea „Mineralogia Magni Principatus Transsilvaniae”.

Au fost publicate monografii despre Ţinutul Secuiesc, deosebit de bogat în ape minerale şi mofete, prima în 1869 a lui O. Balázs la Pesta în 6 volume, care includ în volumul III prezentarea apelor minerale din jud. Odorhei, ca şi a solfatarei de la Toria, cu analize chimice şi indicaţii de utilizare, a doua a lui W. Hankö în 1890 la Budapesta despre „Băile şi stabilimentele cu ape minerale din jud. Ciuc”.

“Mofetele sunt emanații naturale de dioxid de carbon uscat a cărui concentrație depășește în general 90%, alături de cantități reduse de neon, argon, heliu și altele asemenea. 

Caracterul de gaz mofetic este atribuit și dioxidului de carbon provenit în urma procesului de decarbogazeificare naturală a apelor minerale naturale - natural carbogazoase în timpul lucrărilor de extracție a acestor ape”.

Emanația exclusiv naturală de gaz, provenind din manifestările tardive postvulcanice, în care alături de CO2, compuși gazoși ai sulfului, în principal H2S, realizează concentrații de 0,05-0,5 mg/m3 este denumită “solfatară”; “amestecurile mixte de gaze carbonice și sulfuroase rezultate prin emanații simultane exhalativ postvulcanice, sunt numite “gaze mofeto-solfatariene”, acestea sunt în mofetele de la Turia (0,56 mg/%) și Băile Harghita (0,08 mg%), situate la altitudine, unice în Europa (N. Teleki, L. Munteanu, 2012). 

Emanațiile naturale de CO2 din zona Carpaților Răsăriteni, legate de vulcanismul din munții Gutâi-Căliman-Harghita reprezintă una din bogățiile naturale importante ale României, cu eficacitate terapeutică, valorificată în mod original prin amenajarea unor instalații speciale. În stațiunile balneo-climatice dezvoltate în această zonă: Covasna, Bálványos, Băile Tușnad, Sângeorz Băi, Băile Harghita, Miercurea Ciuc, sunt valorificate surse bogate cu concentrații între 94 – 99% CO2. Asemenea emanații de gaz bioxid de carbon există și în zone din munții Apuseni, cu conținut variabil de CO2 (4 – 81%) și cu conținut mare de azot (18 – 91%). În unele stațiuni cu ape carbogazoase este utilizat gazul CO2 extras din apele minerale și folosit ca mofete, denumire extinsă și la procedurile ce folosesc CO2 industrial – mofete artificiale (dupa N. Teleki, L. Munteanu, 2012). Mofete se mai găsesc în Italia, Franţa, America și mai rar în alte țări.

În funcție de tipul de alimentare, în mofete, acestea sunt:

- pe sursă, care utilizează gazul direct din emergența naturală;

- alimentate, care utilizează gazul transportat din surse forate sau din alte surse prin intermediul unor rezervoare de înmagazinare și distribuție;

- alimentate cu dioxid de carbon industrial îmbuteliat.

În funcție de gradul de umidificare mofetele sunt:

– Uscate;

– Umede.

În baza cercetărilor știintifice moderne și complexe, după cura cu dioxid de carbon în mofete, au fost constatate efecte însemnate cardio-circulatorii. Cufundarea corpului în gazul dioxid de carbon în amenajări speciale sau “mofetării”, produce “efecte vasodilatatoare asupra tegumentului cufundat, la care se pot adauga și efectele decreștere a circulației sangvine musculare prin CO2 dizolvat în plasma. Efectele sistemice prin scăderea rezistenței periferice și a tensiunii arteriale minime, influențează hemodinamica cordului: scăderea perioadei de preejecție și creșterea perioadei de ejectie, cu scăderea raportului dintre ele. S-au remarcat și creșteri semnificative ale fluxului cerebral” (după N. Teleki, L. Munteanu, 2012).
