# Supplementary material for: A XIV-a Conferință Națională de Balneologie și Recuperare Medicală 07 – 09 Aprilie 2017 U.M.F. „Carol Davila” - Aula Facultății de Farmacie, București
Source: J Med Life. 2017 Apr 7;10(Spec Iss 1):7–36. [Article in Romanian] (PMC6088550)
Supplement: Related texts

# Meeting report: PROGNOSTICUL DE RECUPERARE FUNCȚIONALĂ LA UN PACIENT CU SPONDILITĂ ANKILOZANTĂ ȘI TRAUMATISM VERTEBROMEDULAR TORACAL

**Authors:** Maria Asavei, Liliana Cioc, Horia Lăzărescu

**Affiliations:** * I.N.R.M.F.B. – Clinica Recuperare Medicală-Neurologie IV

Introducere: Spondilita anchilopoetică este o afecțiune sistemică cronică și o boală inflamatorie, cu o dezvoltare preponderent la nivelul scheletului axial, caracteristica majoara a bolii fiind afectarea precoce a articulațiilor sacroiliace, boala evoluând spre anchiloza coloanei vertebrale. Aceștia sunt supuși riscului de fractură, în urma unor traumatisme de intensitate medie sau mică, datorită accentuării cifozei, instalării ulterioare a rigidității și respectiv datorită osteoporozei existente. 

Prezentare de caz: Lucrarea de față își propune să prezinte cazul unui pacient în varsta de 45 de ani, cunoscut cu spondilită anchilozantă formă axială cu sacroileită bilaterală stadiul IV, antigen HLAb27 pozitiv, diagnosticată în 1993 și neglijată terapeutic până în 1999 când s-a inițiat tratament medicamentos cu Sulfasalazina 2g/zi timp de 6 luni fără ameliorarea simptomatologiei, ulterior s-a instituit tratament cu AINS ( Diclofenac 150mg/zi, Meloxicam) și AIS (infiltrații paravertebrale cu Diprophos) cu eficacitate scăzută. Din 1.07.2013 până în septembrie 2015 a urmat tratament cu Ac monoclonal anti-TNF-α (Adalimumab) în doză de 40 mg la 2 săptămâni. In prezent urmează tratament cu AINS (Dicloreum 150mg/zi) în doză unică. Boala evolueaza în timp cu afectarea șoldului stâng, pacientul dezvoltând o coxită stangă, iar în iunie 2012 se intervine chirurgical practicându-se artroplastie cu proteză totală necimentată de șold stâng. In Septembrie 2015 a suferit o cadere de la propriul nivel soldată cu TVM localizat la nivelul T10-T11, cu deficit motor paraparetic spastic 1/5 BMRC, nivel de sensibilitate T10, tulburări sfincteriene de tip retenție urinară, RCP în extensie bilateral, ROT vii pe membele inferioare, clonus plantar prezent. Examenul RMN: stenoză severă de canal vertebral toracal T10-T11, cu hipersemnal de contuzie centromedulară T10. Se decide intervenția chirurgicală și se practică decompresia posterioară prin recalibrarea canalului vertebral toracal. Postoperar evoluția a fost lent favorabilă, cu recuperarea partială a deficitului motor (3/5 BMRC) . S-a tentat ablația sondei vezicale dar dupa 8 ore în absența reluării micțiunilor se remontează cateter vezical. A urmat cure repetate de recuperare în clinica noastră cu evoluție lent favorabilă prin ameliorarea spasticității și reducerea spasmelor musculare ca intensitate și frecvență, reluarea mersului pe distante scurte-medii cu sprijin în cadru și controlul afecțiunilor asociate (infecții de tract urinar și boala de fond). De menționat că tratamentul cu Adalimumab a fost întrerupt la indicația medicului reumatolog după apariția leziunii medulare și infecțiilor de tract urinar survenite pe fondul noului status neurologic, infecțiile active reprezentând o contraindicație majoră pentru terapia biologică. Pacientul a fost internat în clinica noastră pentru deficit motor paraparetic, durere cu caracter inflamator la nivelul genunchiului drept, tulburări sfincteriene- disinergie vezico-sfincteriană, deficit de locomotie și autoîngrijire sever. 

Management și rezultate: Obiectivele tratamentului fizical-kinetic în acest caz au fost următoarele: controlul afecțiunilor asociate, prevenirea și combaterea complicațiilor, combaterea durerii și inflamației, creșterea capacității aerobe și reeducare respiratorie, îmbunătățirea statusului funcțional, reintegrare socio-profesională și educația pacientului și a familiei. Pe parcursul internării evoluția a fost favorabilă cu creșterea forței musculare la nivelul trenului superior și inferior, cu ameliorarea spasticității pe membrele inferioare, cu îmbunătățirea funcției respiratorii, cu creșterea toleranței la efort (dacă inițial realiza mersul la REO ambulator (antrenamentul mersului cu feedback vizual și auditiv) timp de 5minute, la externare timpul a crescut la 15 minute), cu ameliorarea simptomatologiei algice. 

Concluzii: Prezentarea unui caz clinic la un pacient în varstă de 45 de ani, care a suferit un TVM pe fondul unei spondilite anchilozante cu sacroileită bilaterală stadiul IV diagnosticată în urma cu aproximativ 23 de ani, leziunea medulara influențând atat tratamentul de recuperare cât și prognosticul functional și pe cel vital. Contrar complicațiilor pe care le asociază pacientul prezintă un tonus psihic bun. 

*Cuvinte cheie: Spondilita anchilozantă, traumatism vertebro-medular*.

# Meeting report: ROLUL ECOGRAFIEI MUSCULO-SCHELETALE ÎN RECUPERAREA MEDICALĂ

**Authors:** Daniela Avramescu, Augustin Dima

**Affiliations:** * I.N.R.M.F.B. – Clinica Recuperare Medicală-Ortopedie și Traumatologie (I)

Ecografia sau ultrasonografia reprezintă înregistrarea grafică a ecourilor ultrasonore reflectate. Cu ajutorul ultrasunetelor și a principiilor de bază ale acestora, se pot vizualiza structurile musculo-scheletale. Obiectivele sunt cunoașterea metodelor de screening, diagnostic, prognostic evolutiv și tratament furnizate de ecografia musculo-scheletală în practica medicală curentă, îndeosebi importanța acestora în recuperarea medicală.

Aparatele de ecografie de ultimă generație dispun de o bună rezoluție, acestea putând de asemenea integra examinarea Doppler, extinderea câmpului imaginii vizualizate în scopul permiterii unei imagini de ansamblu, posibilitatea comparării mai multor imagini în timp real, a măsurării structurilor examinate, vizualizarea și calcularea traiectoriei acului, integrarea structurilor 3D, precum și posibilitatea utilizării elastografiei. Ecografia este o metoda ce permite vizualizarea structurilor în dinamică, în timpul manevrelor de flexie-extensie, de contracție-relaxare musculară, fiind cel mai des folosită cu rol diagnostic, dar și ca metoda de ghidaj în scop intervențional în timp real, în cadrul manevrelor de puncție-aspirație și a infiltrațiilor, putând evidenția structurile în planuri diferite, fluxul sanguin; permițând formularea unui diagnostic rapid și fiind o investigație neiradiantă, nedureroasă, neinvazivă, ce poate fi repetată, fiind accesibilă financiar. Cunoașterea anatomiei ecografice, a particularităților și a vastei patologii ecografice este posibilă doar învățând și exersând, aceasta putându-se dobândi după ani de experiență în domeniu, acesta fiind poate principalul dezavantaj al ecografiei musculo-scheletale.

Aducând un real beneficiu în practica medicală curentă, îndeosebi în medicina de recuperare, unde ne confruntăm frecvent cu problema pacienților neinvestigați și nediagnosticați, pentru care medicul de recuperare este primul la care apelează pentru afecțiuni musculo-scheletale, văd necesitatea cunoașterii și a perfecționării acestei metode de diagnostic și tratament.

# Meeting report: ORTEZAREA STATICĂ ȘI DINAMICĂ A MEMBRULUI SUPERIOR ÎN CONTEXTUL AVC. DE LA CONCEPT TEORETIC LA APLICAȚII PRACTICE ÎN CADRUL REABILITĂRII MEDICALE.

**Authors:** Andrei Bichis, Yulyia Manolaki, Liliana Cioc, Horia Lazarescu

**Affiliations:** * I.N.R.M.F.B. – Clinica Recuperare Medicală-Neurologie (IV)

Lucrarea de față își propune să evidențieze rolul primordial pe care îl are ortezarea statică și dinamică în recuperarea medicală a membrului superior.

Pe parcursul acestei prezentări au fost prezentate și alte metode și mijloace auxiliare care și-au adus aportul la reabilitarea membrului superior și implicit la promovarea cât mai aproape de normal a motilității pasive și ulterior a motilității active. 

Cele mai eficiente metode de recuperare uzitate au fost pe lângă ortezare următoarele;

• terapia ocupatională

• mobilizările pasive și active

• metodele Kabat, Bobath și Le Metayer

• terapia cu nisip kinetic

• elemente ludice inspirate din terapia de loisir și recreațională

Scopul tratamentului fiziatric a fost să amelioreze controlul motor la nivelul membrului superior afectat de AVC.

Pacienții au urmat un tratament de recuperare medicală suplimentară constând într-o ședință de 50 min/zi cu pauză la fiecare 5 min de 30 sec uneori de un min timp de două săptămâni, pauza a fost de tip odihnă activă în acest scop s-au folosit și metodele de relaxare Jacobson și Schultz. Controlul motor al acestor pacienți s-a ameliorat semnificativ acestea reușind să integreze în schema corporală și miscările efectuate cu membru superior.

Pe viitor ne propunem să îmbunătățim ortezarea membrului superior prin folosirea de mijloace terapeutice specifice recuperării medicale și de asemenea prin fabricarea personalizată și adaptată a diferitelor tipuri de orteze pentru fiecare pacient ținând cont de patologia asociată și de statusul psihic al acestora.

# Meeting report: TRATAMENTUL DE RECUPERARE ÎN DISFAGIA NEUROGENĂ

**Authors:** Ivona Burlacu, Dan Dumitrașcu, Delia Cinteză, Horia Lăzărescu

**Affiliations:** * I.N.R.M.F.B. – Clinica Recuperare Medicală-Neurologie (IV)

Disfagia sau dificultatea la deglutiție este definită ca afectarea tranzitului bolului alimentar din cavitatea bucală în faringe şi esofag. Tulburarile de deglutiție au un impact negativ marcant asupra calității vieții și sunt o cauză importantă de morbiditate şi mortalitate prin potențialele complicații pe care le pot dezvolta, cea mai importantă fiind reprezentată de aspirația de fragmente din bolul alimentar în caile respiratorii, cu toate consecințele acesteia. Disfagia poate avea cauză neurogenă sau non-neurogenă. Tratamentul necesită atât regim igieno-dietetic adecvat, cât și medicamentos, dar, mai ales, de recuperare.

# Meeting report: MANAGEMENTUL SPASTICITĂȚII - METODE DE TRATAMENT FARMACOLOGICE ȘI NON-FARMACOLOGICE

**Authors:** Liliana Cioc, Delia Cinteză, Simona Ioana Neagoie, Irina Petrușca, Victorița Suciu, Elena Ganea, Andrei Bichis, Horia Lăzărescu

**Affiliations:** * I.N.R.M.F.B. – Clinica Recuperare Medicală-Neurologie (IV)

Introducere: Spasticitatea este o componentă a sindromului de neuron motor central. Spasticitatea reprezintă de multe ori o problemă interferând cu recuperarea și aducând pacientului inconveniente și complicații. Managementul spasticității se bazează pe metode de farmacoterapie, fizioterapie și intervenții neuro-chirurgicale. Evaluarea clinică detaliată și evaluarea funcțională sunt necesare deoarece spasticitatea nu are nevoie întotdeauna de tratament, în special atunci cand nu există niciun obiectiv realist de câștig funcțional. Pentru combaterea spasticității este necesar să se identifice și să se înlature orice stimul nociceptiv.

Obiective: Prezentarea metodelor de tratament farmacologice și non-farmacologice ce se utilizează la momentul actual în combaterea spasticității, insistând în mod special pe aspecte legate de eficiență și siguranță, indicații și contraindicații.

Material și metodă: Informații din studii clinice internaționale, ghiduri de tratament și date din experiența practică a clinicii cu privire la mecanismul de acțiune, indicații, doze, eficiența și riscuri ale tramentului.

Rezultate: Obiectivele tratamentului sunt reprezentate de: îmbunătățirea ADL-urilor, ameliorarea mersului, înlăturarea durerii, combaterea ankilozelor articulare, creșterea stabilității și a mersului. Principalele metode fizical-kinetice cu rol în ameliorarea spasticității sunt reprezentate de posturare, stretching, crioterapie, hidroterapie, termoterapie, mișcare asistată, inclusiv FES, ortezare, biofeedback EMG, tehnici FNP și masaj sedativ. Tratamentul medicamentos oral cuprinde baclofenul, bezodiazepinele, agoniștii alfa 2, gabapentinul și dantrolenul sodic. Agentul farmacologic este ales în funcție de severitatea spasticității, distribuția anatomică, probleme medicale concurente și statusul cognitiv. Pentru spasticitatea focalizată, foarte eficiente pot fi infiltrațiile cu toxină botulinică.

Concluzii: Spasticitatea este o manifestare frecventă în patologia neurologică, care poate afecta calitatea vieții unui pacient și interfera cu funcționalitatea. Spasticitatea nu este în mod necesar inamicul, dar este parte a unui pattern anormal al controlului motor. Managementul spasticității poate fi uneori dificil. Tratamentul spaticității trebuie individualizat în funcție de fiecare pacient, de simptomatologia clinică.

# Meeting report: INCERTITUDINEA DE MĂSURARE A INDICATORILOR FIZICO-CHIMICI DIN APELE MINERALE TERAPEUTICE

**Authors:** Mădălina Cosmoiu, Liana Gheorghievici, Gh. Gheorghievici, Margareta Mustață, Iosif Tănase, Iulia Pompei

**Affiliations:** * I.N.R.M.F.B. – Departament Cercetare - Laborator de Încercări Ape Minerale și Nămoluri Terapeutice

Una din cerințele fundamentale puse în fața laboratoarelor de încercări conform standardului ISO 17025 o constituie estimarea și raportarea bugetului de incertitudine precum și asigurarea trasabilității la etaloanele internaționale.

Standardul SR EN ISO/CEI 17025:2006 pune mai mult decît precedentele sale accentul asupra conceptului de incertitudine în domeniul de încercări, asupra evaluării sale, menţiunea în rapoartele de încercări şi utilizarea cu scopul declarării conformităţii.

Incertitudinea de măsurare este un parametru asociat cu rezultatul unei măsurări, care caracterizează dispersia valorilor ce pot fi atribuite în mod rezonabil masurandului. Importanţa incertitudinii de măsurare pentru utilizarea şi interpretarea rezultatelor încercărilor sau etalonărilor este cu adevărat indiscutabilă, paralel este necesar de subliniat că identificarea şi urmărirea componentelor de incertitudine contribuie larg la însuşirea metodelor de încercări şi etalonări.

Atunci când se estimează incertitudinea de măsurare trebuie luate în considerare toate componentele de incertitudine care au importanță în situatia dată, folosind metode de analiză adecvate. Laboratorul I.N.R.M.F.B. de Incercări Fizico-Chimice și Microbiologice Ape Minerale și Nămoluri Terapeutice are și aplică proceduri pentru estimarea incertitudinii de măsurare. In anumite cazuri natura metodelor de încercare poate să nu permită un calcul riguros, metrologic statistic valid al incertitudinii de măsurare. In aceste cazuri laboratorul trebuie cel putin să încerce să identifice toate componentele de incertitudine și să facă o estimare rezonabilă.

Absenţa referinţei la incertitudine în rapoartele de încercări sau utilizarea acestor incertitudini de măsurare pentru a face o declaraţie de conformitate sau a interpreta rezultatele nu scuteşte laboratorul de satisfacerea cerinţei precedente. 

Evaluarea incertitudinii de măsurare pentru încercări cantitative aplica legea compoziţiei variaţiilor, a utilizarii fidelităţii şi justeţii metodelor de încercări sau de analiză, determinarea liniarităţii cu ajutorul materialului de referinţă sau utilizarea datelor testelor de competenţă.

Calculul incertitudinii de măsurare nu este niciodată o misiune de rutină și nici o operație pur matematică; aceasta depinde de cunoașterea temeinică a naturii masurandului și a măsurării.

Calitatea și utilitatea incertitudinii stabilite pentru rezultatul unei măsurări depind în ultimă instanță de analiza critică, de onestitatea intelectuală și de componenta profesională a celor ce contribuie la evaluarea ei.

# Meeting report: TRATAMENT RECUPERATOR ÎN COAFECTĂRILE MUSCULO-SCHELETALE LA UN PACIENT CU DISFUNCȚIE MIOTONICĂ STEINERT

**Authors:** Gheorghe Chiriti, Dana-Maria Dimulescu, Rodica Gabriela Scarlet, Vasile Cîrlig

**Affiliations:** * I.N.R.M.F.B. – Clinica Recuperare Medicală-Ortopedie și Traumatologie (I); ** I.N.R.M.F.B. – Clinica Recuperare Medicina Fizică și Balneologie (II)

Obiective: Scopul acestei prezentări de caz este de a evidenția rolul programului complex fizical-kinetic, în recuperarea coafectărilor musculo-scheletale, la un pacient cu distrofie miotonică Steinert.

Material și metodă: Vă prezentăm cazul unui pacient de 39 ani, internat în clinica noastră pentru sindroame algo-disfuncționale multiple la nivelul membrelor superioare, membrelor inferioare, ca rezultat al unei tulburări de contracție musculară, caracterizată printr-o decontracturare anormal de lentă, cu dificultatea revenirii la starea de relaxare completă, impotență funcțională- mers dificil, cu sprijin auxiliar, cu viteză de deplasare lentă, cu deplasarea centrului de greutate spre anterior, prin tulburari compensatorii (flexum de șold, flexum de genunchi). 

Din antecedentele patologie evidențiem: frecvente traumatisme prin cădere de la propria înălțime, soldate cu afectări ale parților moi, fără lezarea țesutului osos.

La examenul local: sindrom vertebral static și dinamic la nivel CDL, cu accentuarea cifozei toracale, hiperlordoza lombară, contractură musculară paravertebrală toraco-lombară, cu reducerea mobilității active dorso-lombare și lombo-pelvine, cu scăderea indicilor Ott, Schober, Macrae-Wright; reducerea mobilității active pe toate axele de mișcare șold-genunchi-complex glezna-picior, cu hipotrofii coapse, gambe bilateral; modificarea schemei de mers prin deplasarea centrului de greutate spre anterior, mers cu pasi mici, cu viteză lentă de deplasare, cu alterarea fazei de inițiere a mersului, cu sprijin auxiliar.

Programul complex recuperator a inclus: tratament antialgic și decontracturant, electroterapie cu efect antialgic și miorelaxant (TENS), termoterapie (laser, ultrasunet, parafină); masaj cervico-dorso-lombar, cu efect decontracturant pe musculatura paravertebrală, alternativ cu masaj mixt (sedativ-tonizant) la nivelul musculaturii membrelor pelvine; kinetoterapie la sală axată pe: exerciții de asuplizare a musculaturii paravertebrale cervico-dorso-lombare, exerciții de asuplizare a musculaturii extensoare la nivelul coapsei, genunchiului, exerciții de reeducare a mersului.

Rezultate: In urma tratamentului recuperator aplicat, s-au constatat: ameliorarea parametrilor de mers (viteză de mers, lungimea pasului), ameliorarea stabilității în timpul mersului, îmbunătățirea posturii la nivel CDL.

Concluzii: Programul recuperator fizical-kinetic a ameliorat sindromul algo-disfuncțional de la nivelul trenului inferior, respectiv CDL; ameliorări notabile s-au înregistrat după a doua internare în clinica noastră, la interval de 5-6 luni de la internarea inițială, pacientul reușind pe distanțe scurte-medii să se deplaseze fără sprijin auxiliar.

# Meeting report: BENEFICIILE ASOCIERII HIALURONAT DE SODIU 60MG/3ML + CONDROITINĂ SULFAT DE SODIU 90MG/3ML, SOLUŢIE INJECTABILĂ ÎN SERINGĂ PRE-UMPLUTĂ IN TRATAMENTUL OSTEOARTRITEI DE GENUNCHI

**Authors:** Augustin Dima

**Affiliations:** * I.N.R.M.F.B. – Clinica Recuperare Medicală-Ortopedie și Traumatologie (I)

Obiectivul dezvoltării acestui dispozitiv medical a fost acela de a asocia hialuronatului de sodiu, prin asimilarea unui nou produs cu caracteristici funcţionale îmbunătăţite prin asocierea cu sulfatul de condroitină. Acest nou dispozitiv medical de vâscosuplimentare permite gestionarea simptomatică a osteoartritei prin restaurarea proprietăţilor mecanice ale lichidului sinovial deficitar în acid hialuronic.

În general, o varietate de modificări chimice ale acidul hialuronic natural (HA) au fost efectuate pentru a oferi robustețe mecanică și chimică, proprietăți reologice îmbunătăţite şi biostabilitate crescută, păstrând biocompatibilitatea și biodegradabilitatea polimerului nativ. 

Dispozitivul medical destinat administrării intraarticulare, prin conţinutul crescut în hialuronat de sodiu 60mg/3ml (2,0%) de origine non-animală, reticulat natural cu 90mg/3ml (3,0%) condroitină sulfat de sodiu, face posibil să se prevadă o totală biocompatibilitate şi o rată foarte scăzută de evenimente adverse conferite de un produs cu proprietăți fizico-chimice şi mecanice aproape cu cele ale un fluid sinovial uman neafectat de fenomene degenerative, care-l fac optim şi potrivit pentru o vâscosuplimentare sigură și eficientă. 

Mai multe studii au investigat influenţa greutății moleculare (MW) a HA asupra eficacității acestuia ca un tratament intraarticular pentru osteoartrită.

Prin asocierea de condroitină sulfat și hialuronat de sodiu în soluție apoasă, s-a constatat în mod surprinzător că moleculele par să se alinieze și să se atragă reciproc prin legături de hidrogen dezvoltate de gruparea N-acetilamina, interacțiunea prin legături de hidrogen este doar una din mai multele interacțiuni posibile între condroitina sulfat și hialuronatul de sodiu.

Interacția dintre cei doi componenţi este considerată a avea loc la orice concentraţie. Cu toate acestea, efectul sinergic al vascozitătii este mai pronunţat la valori mari ale concentraţiei, datorită efectului de apropiere între cele două molecule. Soluţia rezultată manifestă caracteristici de curgere non-newtoniene şi comportament pseudoplastic.

Solutia apoasă tampon (fosfat mono- şi disodic + clorură de sodiu) este folosit pentru mentinerea pH-ului si osmolaritatii intre 300-350 mOsmoli/Kg. Prin cresterea concentratiei tamponului, creste taria ionica a complexului CS/HA. Totodata, constanta vitezei cinetice a interacțiunii dintre moleculele de CS si HA este crescută prin creșterea tăriei ionice și temperatura. Aceasta se poate explica prin aceea că interacția mutual crește cu creșterea temperaturii datorită vitezei cinetice conducând la o îmbunătățire a mobilității moleculelor pentru alinierea grupărilor N-acetilamina pentru formarea legăturilor de hidrogen intermoleculare.

Derivaţii granulaţi de condroitină sulfat și derivaţii granulaţi de hialuronat sunt capabili de legare şi interacțiune între lanțurile de carbohidrați ale polimerilor. De asemenea, s-a constatat că adăugarea de sulfat de condroitină la hialuronat de sodiu în soluție apoasă sau hialuronat de sodiu la condroitina sulfat în soluție apoasă crește dramatic viscozitatea amestecului. Această creștere a viscozității pare să fie în principal datorită creșterii în greutate moleculară, mai degrabă decât creșterea concentrației solutului. Interacțiunea intermoleculară din soluţie prin legături de hidrogen, între condroitina sulfat și hialuronatul de sodiu conduce la mărirea eficientă a dimensiunii moleculare. 

S-a demonstrat de asemenea, schimbarea neliniară sau sinergică a proprietăților fizice ale amestecurilor celor 2 componente. Prin date de rezonanță magnetică nucleară (RMN) si SEM se susţine ca asocierea CS cu HA are loc prin legături hidrofobe, care se formează între unitatile extinse (9 CH-unități) şi legaturile hidrofobe care se repetă de-a lungul catenei lanțurilor alternative ale polimerului. În acelasi timp, datele de vascozimetrie și cromatografie confirmă interacția dintre cele două molecule. 

Caracterizarea hidrogelurilor a avut drept scop analiza compoziţională şi structurală a materialelor sintetizate prin: analiza elementală, teste reologice în regim vascozimetric si oscilatoriu, determinari ale dimensiunii particulelor si potentialului Z. Studiul examinarii proprietatilor vâscozimetrice şi vascoelastice ale lichidului sinovial normal din articulatia genunchiului uman la diferite viteze de forfecare respectiv, la frecvente diferite în regim vîscoelastic dinamic, corespund diferitelor viteze ale articulatiei. 

În cazul conţinutului de grupe carboxilice din copolimeri, gradul de umflare al hidrogelurilor creşte cu creşterea pH-ului mediului, datorită repulsiilor electrostatice dintre formele ionizate ale grupelor carboxilice. Diferenţele în gradul de umflare pot fi explicate pe seama pH-ului: în mediu acid grupările –COOH ale copolimerilor sunt nedisociate iar în mediu bazic sunt disociate. În stare disociată, au loc repulsii electrostatice între grupele carboxilice anionice, determinând distanţarea acestora şi creşterea volumului ocupat de moleculele de apă, fenomenul conduce la un grad de umflare mai mare.

Comportamentul hialuronatului de sodiu în soluţie apoasă depinde de conformaţia lanţului polimeric care este influenţat de masa moleculară, polidispersitate şi compoziţia chimică unică a moleculei. 

În medii concentrate, prezenţa interacţiilor intra-moleculare (în special al moleculelor cu greutate moleculară mare), afectează dimensiunea ghemului polimeric. Molecula condroitinei sulfat în interacţie cu NaHA va influenţa conformaţia şi dimensiunea ghemului polimeric. Lanţurile hialuronatului pot forma o conformaţie expandată comportament tipic unui polielectrolit anionic flexibil. 

Hidrogelurile sunt reţele tridimensionale care înglobeaza cantitati mari de fluide. Retinerea apei se datoreaza grupelor hidrofile: carboxil si hidroxil (COOH, OH) ce permit formarea legăturilor de hidrogen si a interactiilor hidrofobe (intre lanturile –alchil ale moleculei), care le asigura consistenta. Proprietătile mecanice se îmbunatatesc prin reticulare cu agenti fizici sau chimici. Reticularea conduce la stabilizarea unei retele permanente iar mobilitatea intrinseca a moleculelor scade dramatic. Reţeaua este stabilizată prin legături de hidrogen.

Reticularea fizică este definită prin crosslink non-covalent, dar cu o puternică forță de atracție secundară între lanțurile polimerice. Hidrogelul fizic/reversibil este numit „reversibil“ sau gel „fizic“ atunci când reţelele sunt menţinute împreună prin aglomerări moleculare şi /sau forţe secundare, inclusiv ionice, legături de hidrogen sau interacţiuni hidrofobe. În gelurile reticulate fizic, dizolvarea este prevenită prin interacțiuni fizice, care există între diferitele lanțuri polimerice. Toate aceste interacțiuni sunt reversibile și pot fi perturbate de schimbările în condițiile fizice sau aplicarea de stres. 

Hidrogelurile chimice/ permanente sunt formate prin reticulare covalentă a polimerilor. În ultimii ani, o modalitate comună de a crea o rețea reticulată covalent este de a polimeriza macromeri end-funcționali.

Studiile brevetate susțin asocierea în soluţie apoasă prin legaturi de hidrogen între cei doi componenți. Formarea complexului între HA:CS în raport de 1:1 a fost demonstrată prin date spectroscopice FTIR, potențial Zeta, spectrometria de absorbție UV-Vis. Se prezintă schimbările comformaționale pe care le prezintă complexul format comparativ cu componentele individuale. Ca și în cazul nostru, analizele de potențial Zeta au fost efectuate pentru a caracteriza sarcina suprafeţei complexului format atât fizic cât şi chimic. Rezultatele experimentale arată că potențialul Zeta al amestecului fizic este mai mic comparativ cu cel al amestecului consolidat, confirmând că sunt entităţi diferite structural, cu sarcini de suprafață diferite. Complexul HA:CS se prezintă o rețea extrem de densă formată în urma interacției catenelor polimerului, în comparație cu amestecul fizic simplu, care prezintă o structură dezordonată. 

Soluția de sulfat de condroitină/amestec hialuronat de sodiu prezintă nu numai proprietăţi vâscoelastice dar evidenţiază şi uşor comportament reopectic (de exemplu, vâscozitate crește cu timpul, la viteza de forfecare constantă). Atât sulfatul de condroitină cât şi hialuronatul sunt molecule elicoidale cu lanțuri drepte, în medii extrem de vâscoase la temperaturi reduse, ambele molecule au o mobilitate mai scăzută în alinierea grupărilor N-acetilamină pentru interacțiunea reciprocă, interacțiune care are loc într-un ritm foarte lent. Creşterea temperaturii conduce la intensificarea interacțiunii reciproce ca urmare a creșterii ratei cinetice și o mai bună mobilitate a moleculelor în a-şi alinia grupurile N-acetilamină pentru legături de hidrogen. Când amestecul este supus la o viteză mare de forfecare, moleculele sunt aliniate sau orientate pentru interacțiune-lipire, astfel că vâscozitatea creşte când amestecul este supus la viteză mare de forfecare, și/ sau creșterea temperaturii, rezultând un compus modificat, cu o greutate moleculară mare. 

Vâscosuplimentarea este un tratament sigur, eficient și bine stabilit al osteoartritei și constă în injectarea unei soluţii pe bază de acid hialuronic (HA) în articulațiile afectate. Terapia comună de vâscosuplimentare este concentrată în principal pe tratarea simptomelor, cum ar fi reducerea durerii, dar nu tratează cauza. Cu toate acestea, un tratament optim de vâscosuplimentare ar trebui să întârzie degenerarea cartilajului și chiar să ajute la regenerarea structurii cartilajului. O varietate de modificări chimice aduse acidul hialuronic natural (HA) au fost efectuate pentru a oferi robustețe mecanică și chimică, proprietăți reologice îmbunătăţite şi biostabilitate crescută, păstrând biocompatibilitatea și biodegradabilitatea polimerului nativ.

Astăzi, o familie destul de mare de produse pe bază de acid hialuronic cu diferenţele structurale, precum şi funcţionale, este disponibilă sub forma unor dispozitive de vâscosuplimentare pentru gestionarea simptomatică a osteoartritei de genunchi şi şold. Analiza comparativă a specificaţiilor tehnice şi în special a caracteristicilor reologice ale celor mai relevante produse de vâscosuplimentare din piaţă, arată că acidul hialuronic natural de origine non-animală este mult utilizat dar și că acesta pe lângă reticularea chimică poate fi reticulat şi pe cale naturală. Toate procesele de reticulare chimică, folosesc ca agenţi de reticulare substanțe toxice precum formaldehidă şi divinilsulfonă sau BDDE (1.4-butandiol diglicidileter). Originea non-animală şi reticularea naturală cu sulfat de condroitină face posibil să se prevadă o totală biocompatibilitate şi o rată foarte scăzută de evenimente adverse conferite de un produs cu proprietăți fizico-chimice şi mecanice aproape cu cele ale un fluid sinovial uman neafectat de fenomene degenerative, care-l fac optim şi potrivit pentru o vâscosuplimentare sigură și eficientă. 

Hialuronatul de sodiu este un polizaharid cu masă moleculară mare, un glicozaminoglican compus din unităţi de glucuronat de sodiu şi N-acetilglucozamină, un biopolielectrolit anionic, caracter datorat grupărilor carboxil (COO-H+) ale unităţilor acidului glucuronic. Fiecare unitate dizaharidică are o masă moleculară de 400 Da şi o lungime de ~1,98 Å. Comportamentul hialuronatului de sodiu în soluţie apoasă depinde de conformaţia lanţului polimeric care este influenţat de masa moleculară, polidispersitate şi compoziţia chimică unică a moleculei. Conformaţia în mediul diluat (< 1%) este condusă prin legaturi de hidrogen ce acţionează între moleculele de solvent şi cele ale polizaharidei. Pe masură ce soluţia se concentrează, au loc interacţii inter- şi intra-moleculare. Molecula hialuronat de sodiu formează o structură secundară ordonată şi conţine domenii hidrofobe. Din punct de vedere fizic, domeniile hidrofobe sunt prezente în catena polimerului, în timp ce, grupările de natură hidrofilă sunt localizate în situsurile ramificate ale moleculei. În medii concentrate, prezenţa interacţiilor intra-moleculare (în special al moleculelor cu greutate moleculară mare), afectează dimensiunea ghemului statistic polimeric. 

*Condroitina sulfat* este unul din glicozaminoglicanii naturali (GAG) constituită din zaharuri alternative de D-acid glucuronic şi N-acetil-D-galactozamină. Reprezintă un important component al matricei extracelulare şi cea mai frecventă GAG în molecula *agrecan* a cartilajului. Ph. Eur. 7.3 defineşte sarea de sodiu a sulfatului de condroitină drept un copolimer natural compus în principal din două dizaharide: acid [4)-(β-D-glucopiranosiluronic acid)-(1->3)-[2-(acetilamino)-2-deoxi-β-D-galactopiranosil 4-sulfat]-(1->] şi acid [4)-(β-D-glucopiranosiluronic acid)-(1->3)-[2-(acetilamino)-2-deoxi-β-D-galactopiranosil 6-sulfat]-(1->] sare de sodiu, care la o hidroliză completă eliberează D-galactozamina, acid glucuronic, acid acetic şi acid sulfuric.Condroitina sulfat se obţine din cartilaje de origine terestră sau marină. În funcţie de originea speciilor animale din care se extrage, prezintă proporţii diferite de grupe 4-sulfat şi 6-sulfat. Sulfatul de condroitină a fost iniţial izolat înainte de elucidarea structurii, ceea ce a condus în timp la schimbări de terminologie. Cercetările timpurii au identificat prin litere diferitele fracţiuni izolate: 

**Table:** 

Identificare	Poziţia sulfatării	Denumirea sistematică
*Condroitina sulfat A *	C4 al N-acetilgalactosamina (GalNAc)	condroitina-4-sulfat
*Condroitina sulfat C *	C6 al GalNAc	condroitin-6-sulfat
*Condroitina sulfat D *	C2 al acidului glucuronic şi C6 al GalNAc	condroitina-2,6-sulfat
*Condroitina sulfat E *	C4 şi C6 al GalNAc	condroitina-4,6-sulfat

Molecula condroitinei sulfat în interacţie cu hialuronatul de sodiu va influenţa conformaţia şi dimensiunea ghemului polimeric al acestuia, lanţurile de hialuronat pot forma o conformaţie expandată de ghem statistic, comportament tipic unui polielectrolit anionic flexibil cu proprietăţi îmbunătăţite de lubrifiant şi de protecţie mecanică la stres pentru ţesuturile sinoviale şi cartilajul articular.

Auto-asocierea între moleculele de hialuronat de sodiu şi condroitină sulfat de sodiu în mediu apos prezentată în schema alăturată se bazează pe un mecanism de formare de legături de hidrogen (interacţiuni noncovalente de tip intermolecular) şi interacţii de tip hidrofob (stabilite între lanţurile polizaharidice, grupările CH3 şi atomii de H axiali) cu formarea unui complex stabil în soluţie apoasă. 

Auto-asocierea moleculelor hialuronat de sodiu şi condroitina sulfat de sodiu prin legături intermoleculare, în soluţie apoasă.

**Fig. 1:** Auto-asocierea moleculelor hialuronat de sodiu şi condroitina sulfat de sodiu prin legături intermoleculare, în soluţie apoasă

In plus, condroitina sulfat de sodiu creşte producţia de acid hialuronic prin celulele sinoviale umane, ceea ce constituie un efect benefic asupra menţinerii vâscozităţii lichidul sinovial. De asemenea, conform unor studii specifice condroitina sulfat stimulează metabolismul condroicitar, conducând sinteza colagenului şi proteoglicanilor-componente de bază ale noului cartilaj, inhibă enzimele elastazei leucocitare şi hialuronidaza, care se gasesc în concentraţie mare în lichidul sinovial al pacienţilor cu afecţiuni reumatice. În concluzie, tratamentul injectabil intraarticular pe bază de hialuronat de sodiu reticulat cu condroitină sulfat de sodiu s-a dovedit a fi foarte sigur şi eficient pentru pacienţii cu OA avansată la genunchi.

Rompharm a dezvoltat un dispozitiv medical caracterizat de parametrii de reologie îmbunătăţiţi, un produs care să evidenţieze predominant în intervalul de efort (0,5-2,5Hz) o elasticitate mai mare decât vâscozitatea, cu rapoarte la efort minim (0,5Hz) pornind de la 1,2:1 şi crescând către efort maxim (2,5Hz) la min. de 2:1.

## References

[1] Laurent  T.C. (1998). The chemistry, biology and medical applications of hyaluronan and its derivative. sWenner-Gren International Series, Vol 72.

[2] Turley E.A., Austen L., Vandeligt K., Clary C. (1991). Hyaluronan and a cell-associated hyaluronan binding protein regulate the locomotion of ras-transformed cells. , J. Cell.Biol..

[3] Underhill C.B., Green S.J., Comoglio P.M., Tarone G. (1987). The hyaluronate receptor is identical to a glycoprotein of Mr 85,000 (gp85) as shown by a monoclonal antibody that interferes with binding activity. J. Biol. Chem..

[4] Underhill C.B. , Thurn A.L., Lacy B.E. (1985). Characterization and identification of the hyaluronate binding site from membranes of SV-3T3 cells. J. Biol. Chem..

[5] Vitanzo P.C., Sennett B.J. (2006). Hyaluronans: is clinical effectiveness dependent on molecular weight?. Am. J. Orthop.

[6] McCourt P.A., Ek B., Forsberg  N., Gustafson S. (1994). Intercellular adhesion molecule-1 is a cell surface receptor for hyaluronan. J. Biol. Chem.

[7] Aubert, MD Jean-Daniel A new viscosupplement based on naturally cross-linked hyaluronic acid: A comparative study. Department of Orthopaedic Surgery, Clinique St Louis, Poissy, France..

[8] 3.9.Physical crosslinking. Polymer Chemistry, chapter 3 Chemical structures and polymer morphology.

[9] (2014). Cross-linking in Hydrogels - A Review. American Journal of Polymer Science.

# Meeting report: SECHELELE POSTRAUMATICE ALE GENUNCHIULUI; ROLUL KINETOTERAPIEI ÎN RECUPERAREA LEZIUNILOR GENUNCHIULUI

**Authors:** Alexandru Cristea, Augustin Dima

**Affiliations:** * I.N.R.M.F.B. – Clinica Recuperare Medicală-Ortopedie și Traumatologie (I)

Genunchiul este cea mai mare articulaţie portantă a corpului, dar şi cea mai complicată din punct de vedere al structurilor intraarticulare şi periarticulare. Prin poziţia acestuia, ca şi prin slaba sa acoperire cu ţesuturi moi, devine deosebit de predispus şi vulnerabil traumatismelor.( Prof. A. Rădulescu afirma că 1/3 din toate afecţiunile traumatice şi 1/4 din totalitatea plăgilor articulare sunt localizate la genunchi).

Cunoaşterea leziunilor traumatice şi mai cu seamă, rezolvarea integrală şi corectă este deosebit de importantă, pe de o parte pentru a evita apariţia sechelelor şi recidivelor, iar pe de altă parte pentru a asigura reluarea activităţii desfăşurată înaintea traumatismului.

Recuperarea se conduce după unele principii comune indiferent de starea dezvoltării fizice anterioare a pacientului, vârstă şi sex, diferenţierea făcându-se doar de ordin cantitativ şi de execuţie impus în timpul şedinţelor.

Metodologia recuperării trebuie să fie concordantă cu vârsta pacientului, cu nivelul pregătirii fizice anterioară a acestuia precum şi cu forma şi gravitatea leziunii.

Metodele kinetoterapiei introduse în primele zile după producerea traumatismului scurtează perioada de recuperare şi asigură reluarea activităţii într-un timp cat mai scurt.

Selecţionarea metodelor de recuperare trebuie aleasă corespunzător deficitului funcţional şi gravității leziunii traumatice și trebuie să cuprindă trei mari obiective: combaterea durerii (indoloritatea), stabilitatea articulaţiei și mobilitatea articulară.

Obţinerea indolorităţii este un deziderat important realizabil pe cale medicamentoasă şi fizioterapeutică (de competenţa medicului şi a fizioterapeuţilor), dar şi prin repaus articular. Obţinerea indolorităţii este primul obiectiv în recuperarea genunchiului, nu numai pentru că un genunchi dureros este practic nefuncţional (atât pentru susţinere, cât şi pentru mişcare), ci şi pentru faptul că durerea limitează mult posibilitatea de recuperare a elementelor sechelare disfuncţionale postraumatice.

Stabilitatea genunchiului în ortostatism sau în mers este asigurată pe de o parte de aparatul capsulo-ligamentar (stabilitate pasivă), iar pe de alt parte de aparatul musculo-tendinos (stabilitate activă) afectarea unuia sau altuia determină aşa numita „instabilitate a genunchiului”. Recuperarea deficitului stabilităţii poate fi ortopedică sau chirurgicală urmată de o lungă perioadă de recuperare funcțională obținută prin tonifierea musculaturii stabilizatoare a genunchilor și creșterea rezistenței ligamentare.

Atât traumatismele directe, cât şi imobilizarea articulară pentru o fractură sau pentru alte cauze, determină de regulă, limitarea mobilităţii genunchiului. Pentru creşterea amplitudinii flexiei, se vor executa mobilizări pasive și active. Recuperarea flexiunilor este un obiectiv funcţional primordial, deoarece pierderea mobilităţii pe ultimele grade de extensie, privează genunchiul de stabilitatea în mers din momentul sprijinului.

Cele mai bune rezultate recuperatorii se obțin atunci când tratamentul este complex: kinetoterapie asociată cu alte mijloace fizicale, masaj și psihoterapie şi desăvârșit prin cure balneare.

# Meeting report: TRATAMENTUL DE RECUPERARE A PACIENȚILOR CU RUPTURĂ DE TENDON ACHILIAN

**Authors:** Drăghescu Laurenția, Romila Andreea, Scarlet Rodica, Tuțu-Pața Viorel, Dimulescu Dana

**Affiliations:** * I.N.R.M.F.B. – Clinica Recuperare Medicina Fizică și Balneologie (II)

Rupturile de tendon Achilian reprezintă o patologie relativ frecventă în cadrul patologiei traumatice, interesând în special grupe de vârstă active din punct de vedere motor, cu sau fără pregătire fizică specifică și cu un regim de activitate variind de la sedentarism la activitate fizică intensă.

Ruptura tendonului Achilian, parțială sau totală, se situează la nivelul joncțiunii tendinomusculare, mai ales la tineri, sau la 2 cm de inserția pe calcaneu la vârstnici, un rol important având și vascularizația tendonului, zona predispusă injuriei fiind relativ avasculară.

Tratamentul poate fi conservator, în cazul rupturilor parțiale, la pacienții vârstinici și la pacienții cu boli asociate care pot încetini vindecarea. In cazul rupturilor totale singurul tratament este cel chirurgical și constă în sutura simplă a tendonului, în reinserția tendonului sau poate implica chirurgia plastică și reparatorie dacă suturile simple nu reușesc. 

Programul de recuperare după tratamentul rupturii, fie conservator sau chirurgical, reprezintă o etapă importantă în procesul de vindecare și de reluare a funcționalității și activităților anterioare.

# Meeting report: ACCIDENTĂRILE ALERGĂTORILOR DE ANDURANȚĂ

**Authors:** Sebastian Diaconescu, Delia Cinteza, Irina Petrusca, Pavel Ana Maria, Stanciulescu Elena, Petcu Viorel, Iliescu Andrei

**Affiliations:** * I.N.R.M.F.B. – Clinica Recuperare Medicală-Neurologie (IV)

Premize-Incidența anuală a accidentărilor importante pentru alergătorii de anduranță este între 34 si 65 %; cele mai frecvente cauze sunt reprezentate de erorile din timpul antrenamentelor (durata, frecvența , intensitatea , schimbările rapide și/sau tranzițiile în program ); alți factori ce contribuie sunt cei anatomici și biomecanici, pantofii, suprafața de alergat, vârsta , sexul și experiența sportivului; un istoric de accidentare anterioară reprezintă, de asemenea ,un important factor de risc pentru recidivă.

Obiectivul acestui studiu este reprezentat de cuantificarea celor mai frecvente accidentări ce apar la alergătorii de anduranță.

Material și metodă –studiul, aflat înca în desfașurare, este unul prospectiv; pacienții inclusi sunt alergători de cel putin 30 km pe săptămână; sunt înregistrate accidentările acestora la prezentarea în clinică, precum și istoricul personal de accidentări importante, ce au determinat întreruperea activității fizice pentru o perioadă mai mare de 6 săptămâni.

Rezultatele preliminarii permit determinarea cu exactitate a celor mai frecvente probleme cu care se confruntă sportivii.

Concluzii-cele mai frecvente accidentări apărute la alergătorii de cursă lungă sunt reprezentate de durerea anterioară a genunchiului (probleme la nivelul mecanismului extensor), sindrom de tract iliotibial, tendinopatia achileană, sindromul tibiilor stresate, fasciita plantară, fractura de stres.

# Meeting report: TRATAMENTUL FIZICAL-KINETIC ÎN COAFECTĂRI MUSCULO-SCHELETALE LA UN PACIENT CU DISTROFIE MUSCULARĂ PROGRESIVĂ SCAPULO-PERIONIERĂ

**Authors:** Dana-Maria Dimulescu, Gheorghe Chiriți, Rodica Gabriela Scarlet

**Affiliations:** * I.N.R.M.F.B. – Clinica Recuperare Medicina Fizică și Balneologie (II); ** I.N.R.M.F.B. – Clinica Recuperare Medicală-Ortopedie și Traumatologie (I)

Obiective:

Scopul acestei prezentări de caz este de a reliefa importanța tratamentului complex recuperator, la un pacient cu distrofie musculară progresivă scapulo-peronieră.

Material și metodă:

Vă prezentăm cazul unui pacient de 36 ani, internat în clinica noastră pentru sindrom algo-disfuncțional membre brahiale, membre pelvine (stg.>dr., fractură recentă tibio-tarsiană stânga, tratată ortopedico-chirurgical), impotență funcțională severă, cu mers stepat bilateral, cu sprijin în cârjă canadiană, dificultate la trecerea de la clinostatism la ortostatism (necesita ajutor), imposibilitatea mersului pe vârfuri și pe călcâie.

Din antecedetele heredo-colaterale reiese caracterul genetic al bolii: mama, sora, mătușa suferă de aceeași afecțiune.

Din antecedentele personale: boala a debutat în copilărie (aprox.6 ani), cu dificultate la mers, cu traumatisme frecvente prin cădere de la propria înălțime, diagnosticul fiind stabilit în jurul vârstei de 9 ani.

La examenul local: amiotrofii la nivelul membrelor superioare bilateral (distal>proximal), cu afectarea penselor și prehensiunii, ce necesita ajutor în efectuarea ADL-urilor, sindrom vertebral static și dinamic CDL, cu hiperlordoză lombară, contractură paravertebrală bilaterală lombo-sacrată; limitare moderată a mobilității active la nivelul membrelor pelvine, amiotrofii membre pelvine bilateral (distal>proximal); ROT abolite global, RCP indiferent bilateral; mers imposibil pe vârfuri și pe călcâie, schema de mers modificată prin alterarea fazei de inițiere a mersului, scurtarea perioadei de balans; mers stepat bilateral, cu sprijin în cârjă canadiană.

Programul recuperator a constat în: tratament antialgic, electroterapie antialgică și miorelaxantă (IF, unde scurte), electrostimulări pe musculatura distală extensoare mână, picior (bilateral), proceduri vasculo-trofice (BG – 4 celulară), masaj sedativ la nivel CDL, masaj mixt (sedativ-tonizant), membre brahiale, membre pelvine; kinetoterapie la sala incluzând: exerciții de asuplizare CDL, exerciții de tonifiere stabilizatori centura scapulară, pelvină, kinetoterapie posturală la nivelul segmentului axial, membre superioare, inferioare, exerciții de reeducare a mersului, cu sprijin auxiliar.

# Meeting report: REEVALUARE A REZULTATELOR EFECTELOR URODINAMICE ALE STIMULĂRII ÎN REGIM ACUT A NERVULUI TIBIAL POSTERIOR PENTRU VEZICA NEUROLOGICĂ HIPERACTIVĂ

**Authors:** Dan Alexandru Dumitrașcu, Andrei-Manu Marin, Irina Petrușcă, Victorița Suciu, Teona Olariu, Sebastian Diaconescu, Delia Cinteză

**Affiliations:** * I.N.R.M.F.B. – Clinica Recuperare Medicală-Neurologie (IV)

Introducere: Majoritatea raportărilor din literatura de specialitate privitoare la stimularea transcutanată a nervului tibial posterior realizată în regim acut la pacienţii cu vezică neurogenă hiperactivă pentru care s-a efectuat injectare de toxină botulinică la nivelul muşchiului detrusor indică un efect imediat favorabil al acestei proceduri neinvazive, sugerând o alternativă eficientă la opţiunile terapeutice existente.

Materiale şi metodă: Intr-un studiu realizat de Dr. Manu şi colab. în clinica de urologie EvoMed au fost înrolaţi pacienţi cunoscuţi cu traumatism vertebro-medular sau sleroză multiplă care prezentau vezică neurogenă de tip retenţie de urină sau de tip mixt, fiind selectaţi cei care asociau un muşchi detrusor hiperactiv diagnosticat prin studiu urodinamic şi eşec terapeutic anterior la administrarea de medicamente anticolinergice/antimuscarinice. Aceştia au beneficiat ulterior de injectare de toxină botulinică în cel puţin 10 puncte la nivelul muşchiului detrusor şi stimulare transcutanată a nervului tibial posterior.

Rezultate şi concluzii: Comparativ cu datele precizate în literatură, unele dintre rezultatele centralizate pentru cei 22 de pacienţi care au finalizat procedurile de diagnostic şi tratament propuse par a fi aparent paradoxale, indicând totuşi posibilitatea ca efectele stimulării nervului tibial posterior în regim acut după injectarea de toxină botulinică (în sens modulator şi de sincronizare a timpilor vezicali) să fie mai curând dependentă de grupa patologică implicată şi de nivelul leziunii medulare, făcând în orice caz necesară efectuarea de studii mai ample şi pentru subgrupuri mai omogene de pacienţi.

# Meeting report: SCLEROZA MULTIPLĂ. PARAPAREZA SPASTICĂ FORMA SECUNDAR PROGRESIVĂ.

**Authors:** Elena Magdalena Ganea, Liliana Cioc

**Affiliations:** * I.N.R.M.F.B. – Clinica Recuperare Medicală-Neurologie (IV)

Pacientă în vârstă de 45 ani diagnosticată în 2004 cu scleroză multiplă se internează în clinică pentru deficit motor membre inferioare drp>stg, vezică neurogenă și disfuncție de locomoție și autoîngrijire moderată.

Examenul clinic: OTS și asupra propriei persoane, echilibrată pulmonar și cardiovascular, transit intestinal prezent, vezică neurogenă, purtătoare de pampers, fără semne de iritație meningiană.

Examenul NMAK: deficit motor paraparetic, vezică neurogenă, forța muscular scazută global pe membrele inferioare bilateral (FM 2), fără tulburări de sensibilitate, clonus plantar prezent, epuizabil după 2-3 secuse, ROT prezente drp>stg, ROT achilian stâng absent, Babinski prezent bilateral, probele de coordonare nu pot fi evaluate.

Funcțional: transferurile, ADL-urile și poziția șezut scurtat le realizează cu dificultate, mersul cu schema modificată, forfecat, atacul solului cu marginea lateral a plantei cu sprijin pe cadru rolator.

Obiective: ameliorarea controlului motor pe membrele inferioare, îmbunătățirea transferurilor, corectarea schemei de mers, îmbunătățirea statusului funcțional.

Particularitatea cazului: pacientă tânară diagnosticată în 2004 cu scleroză multiplă inițial recurent remisivă, ulterior secundar progresivă din 2011 cu prognostic evolutiv rezervat ținând cont de factorii de risc, stilul de viață, starea fizică și psihică a pacientei. Evoluția fiind imprevizibilă în contexul bolii și a aspectelor sus menționate.

# Meeting report: ACTUALITĂȚI DE TERAPIE BALNEARĂ LA NIVEL INTERNAȚIONAL

**Authors:** G. Gheorghievici, B. Mitoiu, M. Constantinovici, R. Nartea, D. Clantau, A. S. Nica

**Affiliations:** * I.N.R.M.F.B. – Clinica Recuperare Medicina Fizică și Balneologie (III)

Lucrarea își propune realizarea unei analize sistematice literaturii de specialitate privind utilizarea terapiei balneare la nivel internațional, punând în lumină orientările terapeutice actuale de folosire a acestei terapii în diferite segmente de patologie.

Este pus în evidență caracterul interdisciplinar prin care se pot îmbogăți strategiile terapeutice în multiplele categorii de suferințe, fiind exprimată și componenta de prevenție ce asociază o diminuare a posibililor factorilor de risc. 

Potențialul de dezvoltare al terapiei balneare prin prisma registrului profilactic se concentrează preponderent pe asociere unor programe de educație terapeutică a pacientului, structurarea unor strategii de nutriție cu scopul optimizării stilului de viață, combaterii sedentarismului și a surplusului ponderal, precum și asocierea diferitelor forme de terapie cognitiv-comportamentală cu scopul facilitării unei mai bune aderențe, aceste elemente conducând la îmbunătățirea calității vieții la acești pacienți. 

Cuvinte cheie: terapie balneară, interdisciplinaritate, registru profilactic.

# Meeting report: UPDATE REGARDING BALNEARY THERAPY IN THE INTERNATIONAL CONTEXT

**Authors:** G. Gheorghievici, B. Mitoiu, M. Constantinovici, R. Nartea, D. Clantau, A. S. Nica

**Affiliations:** * National Institute of Rehabilitation Physical Medicine and Balneoclimatology Third Clinic

The presentation proposes a systematic review of the literature regarding the use of balneary therapy at an international level, offering a perspective over the actual therapeutic orientations of balneotherapy in different chapters of pathology. 

The interdisciplinary characteristic represents a modality to enrich the therapeutic strategies in multiple diseases, and the preventive value is associated with a diminish of risk factors. 

The development potential of balneary therapy through its prophylactic values is focused on the association of therapeutic education programs, the establishment of nutrition strategies in order to influence the patient’s lifestyle, and also the association of different forms of behavioral therapy so as to improve adherence, all these elements leading to long term improvement of the quality of life in these patients.

Key words: balneary therapy, interdisciplinarity, prophylactic value

# Meeting report: PARTICULARITĂȚI DIAGNOSTICE LA PACIENȚII VÂRSTNICI CU OSTEOARTRITĂ ȘI CARDIOPATIE ISCHEMICĂ

**Authors:** Diana Kamal, Rodica Magdalena Trăistaru, Kamal Constantin Kamal, Otilia Constantina Rogoveanu

**Affiliations:** * Universitatea de Medicină și Farmacie din Craiova

Obiective: Datorită simptomatologiei sărace sau atipice a cardiopatiei ischemice la pacienții vârstnici, cel puțin un sfert din cazuri rămân nediagnosticate. Obiectivul studiului nostru a fost reprezentat de identificarea tuturor pacienților vârstnici diagnosticați cu osteoartrită cu diferite localizări și cardiopatie ischemică, internați pe parcursul unui an în clinica noastră. 

Material și metode: Studiul a fost de tip prospectiv și s-a desfășurat pe o perioadă de un an (ianuarie - decembrie 2016). Au fost incluși în studiu un număr de 485 de pacienți, cu vârste cuprinse între 60 și 75 de ani, diagnosticați cu osteoartrită cu diferite sedii. Evaluarea pacienților a fost realizată prin mijloace clinice și paraclinice (analize de laborator, tehnici imagistice-Rx, CT, IRM, electrocardiogramă, Holter ECG pentru 24 sau 48 de ore, ecocardiografie), consult cardiologic. 

Rezultate: În urma anamnezei au putut fi evidențiate simptomele bolii cardiovasculare ischemice la un număr de 117 pacienți vârstnici diagnosticați cu osteoartrită, 34 de bărbați și 83 de femei. In urma efectuării electrocardiogramei și Holter ECG, am constatat că la un număr de 29 dintre cei 34 de bărbați și la 71 dintre cele 83 de femei ce acuzau simptome specifice cardiopatiei ischemice, au fost puse în evidență modificări electrocardiografice. 

Concluzii: Pacienții vârstnici cu osteoartrită prezintă în general un stil de viață sedentar, cu limitarea activității datorită manifestărilor osteo-articulare și nu pot sesiza debutul patologiei cardiovasculare. Realizarea unei anamneze extinse cu insistarea asupra elementelor ce ar putea indica prezența afecțiunii, precum și realizarea unei evaluări clinice și paraclinice minuțioase sunt esențiale în diagnosticarea, evaluarea corectă și aplicarea unui program de recuperare optim la pacienții vârstnici.

# Meeting report: DIAGNOSIS PARTICULARITIES IN ELDERLY PATIENTS WITH OSTEOARTHRITIS AND CORONARY HEART DISEASE

**Authors:** Diana Kamal, Rodica Magdalena Trăistaru, Kamal Constantin Kamal, Otilia Constantina Rogoveanu

**Affiliations:** * University of Medicine and Pharmacy of Craiova

Objectives: Due to poor or atypical symptoms of heart disease in the elderly, at least a quarter of cases remain undiagnosed. The objective of our study was the identification of all elderly patients diagnosed with osteoarthritis of various localisation and ischemic heart disease, hospitalized during a year in our clinic.

Material and Methods: The study was a prospective one and was conducted over a period of one year (January to December 2016). They were included in the study a total of 485 patients, aged between 60 and 75 years, diagnosed with osteoarthritis in different locations. Evaluation of patients was performed by clinical and laboratory (lab tests, imaging techniques-Rx, CT, MRI, ECG, Holter ECG for 24 or 48 hours, echocardiography), cardiology check-up.

Results: Following the clinical history, symptoms of ischemic cardiovascular diseases have been revealed in 117 elderly patients diagnosed with osteoarthritis, 34 men and 83 women. After performing an electrocardiogram and Holter ECG, we found that a number of 29 of the 34 men and 71 of 83 women who accused symptoms of ischemic heart disease were highlighted electrocardiographic changes. 

Conclusions: Elderly patients with osteoarthritis generally have a sedentary lifestyle, with activity limitation due to manifestations of osteoarthritis and cannot perceive the onset of cardiovascular pathology. An extended clinical history, insisting on elements that could indicate the presence of disease, as well as conducting a thorough clinical and laboratory assessment are essential in the diagnosis, proper evaluation and management of the elderly patients.

# Meeting report: ELECTROSTIMULAREA ÎN TRATAMENTUL VEZICII NEUROGENE

**Authors:** Manea Diana, Victorița Suciu

**Affiliations:** * I.N.R.M.F.B. – Clinica Recuperare Medicală-Neurologie (IV)

Termenul de vezică neurogenă înglobează tulburările de stocare și evacuare vezicală, apărute după lezarea parţială sau completă a integrităţii centrilor și căilor nervoase la nivel superior centrului micțiunii de la nivelul S2-S4, corespunzând vertebrelor T12-L1, sau periferic. Astfel, se întâlnește în leziuni sau infecții medulare, scleroză multiplă, accidente vasculare, tumori, sindrom Parkinson, boala Alzheimer sau alte demențe etc. Leziunile situate deasupra acestui nivel antrenează spasticitatea, iar cele situate sub acest nivel determină paralizia flască.

Intrucât disfuncția vezicii urinare cauzează un declin important al calității vieții, au fost dezvoltate mai multe direcții terapeutice, care includ tratament medicamentos, tehnici de electrostimulare și tratament chirurgical.

Obiectivele tratamentului sunt asigurarea unei presiuni scăzute în vezica urinară în timpul umplerii precum și al golirii pentru a preveni deteriorarea aparatului urinar superior, iar prin golirea completă a vezicii urinare se ajută la prevenirea infecţiilor urinare.

Electrostimularea constă în aplicarea unor stimuli electrici nedureroși care produc inhibarea contracției vezicii și stimulează contracția sfincterului vezical extern și a ridicatorului anal. Tehnicile folosite sunt:

• Stimularea nervului tibial posterior - Stimularea electrică nervoasă transcutanată

• Stimularea rădăcinilor sacrale anterioare - produce o reducere semnificativă a volumelor reziduale și riscului de ITU

# Meeting report: RECUPERAREA MEDICALĂ ÎNTR-UN CAZ DE SINDROM DE CANAL LOMBAR ÎNGUST

**Authors:** Yulyia Manolaki, Dan Alexandru Dumitrașcu, Horia Lăzărescu

**Affiliations:** * I.N.R.M.F.B. – Clinica Recuperare Medicală-Neurologie (IV)

Pacient în vârstă de 64 ani cunoscut cu lombalgie cronică, debut ~ 6 ani în urmă, ulcer duodenal operat (~2000), stenoză de canal lombar, anterolistezis L4/L5, HDL2-L5 etajate cu radiculopatii L2-L3 stg și L3-L4, L4-L5 bilateral, sdr. algo-parestezic MI bilateral, coxartroză bilateral, se prezintă pentru durere acutizată la mersul și ortostatismul prelungit, ameliorată în șezut, durere însoțită de parestezii plantare cu caracter ascendent “în șosetă” bilateral.

Examenul obiectiv la internare: OTS și asupra propriei persoane, mucoase și tegumente normal colorate, ganglioni superficiali nepalpabili, echilibrat cardiorespirator, TA=150/100 mmHg, AV=76 b/min, abdomen mobil cu respirația, nedureros spontan și la palpare, cicatrice veche pe linia albă supraombilical până la procesul xifoid cu o ușoară herniere a peretelui abdominal de partea dreaptă, tranzit intestinal păstrat, diureză prezentă, fără semne de iritație meningiană.

Examen NMAK:

Coloana Vertebrală 

Sdr. vertebral static: hiperlordoză cervicală, scolioza toracală dextroconcavă, rectitudinea coloanei lombare. sdr. vertebral dinamic: anteflexia – limitată: Indicele Schober 2 cm, indicele deget sol sub genunchi – 1/3 medie a tibiei, inflexiunile laterale posibile, extensia imposibilă. 

Sdr. radicular: manevra Laseque negativă bilateral, ROT rotuliene egale bilateral, ROT tendon Achilian absente bilateral, RCP în felxie. sdr. musculoligamentar: contractură paravertebrală bilaterală dorso-lombară mai pronunțată pe partea dreaptă, puncte trigger paravertebral lombar și sacral. sdr. dural: absent. sdr. psihoemoțional: moderată anxietate.

Șolduri bilateral: ușoară limitare a rotației la nivelul art.coxofemurale bil. mai ales pentru rotația internă, mișcări cu amplitudine normală în celelalte planuri de mișcare, fără tulburări de forță musculară.

Genunchi bilateral: fără semne de inflamație locală, cracmente la mobilizate, șoc rotulian absent, fără semne de instabilitate antero-posterioară sau latero-laterală, mobilitate și forță musculară bună.

Fără scadere de forță musculară în teritoriile implicate, fără tulburări de sensililitate profundă, cu hipoestezie “în șosetă” MI bilateral mai accentuată pe fața laterală gambă dreaptă. Prezintă fenomene claudicativa de natură neurogene.

Funțional: FIM- 7, independent, mers cu pași mărunți, atacul solului cu talonul, cu antiflexia trunchilui, fără sprijin în dispozitive auxiliare. Mers posibil pe călcâi și vârful piciorului.

In urma examenului clinic și paraclinic se susțin următoarele diagnostice:

• Stenoza de canal lombar 

• Sindromul Fatetal 

• HDL2-L5 etajate cu radiculopatii L2-L3 stg și L3-L4, L4-L5 bilateral

• Anterolistezis L4/L5

• Polineuropatie mixtă predominant senzitivă formă axonală 

• Coxartroză bilaterală 

• HTA std. I ( JNC 8)

• Dislipidemie cu hipercolesterolemie 

• Obiective:

• Combaterea durerii și a inflamației 

• Relaxarea contracturii musculare paravertebrale 

• Restabilirea staticii și a dinamicii coloanei vertebrale 

• Antrenare mers 

• Imbunătățirea statusului funcțional

Pacientul a urmat în clinică tratament medicamentos (Dexametazonă 1fi/zi i.m., Pentoxi Retard 400 mg 1-0-1, Omez 20 mg 1-0-0, Rimalaya Forte 1-0-0, Liv 52 1-0-0, Prozac 20 mg 1-0-0, Mydocalm 150 mg 1-0-1, Noliprel ARG forte 1-0-1) tratament de specialitate KT pentru asuplizare CDL, tonifiere stabilizatori abdomen, tonifiere musculatură stabilizatorilor art. CF bilateral, antrenamentul mersului, creșterea toleranței la efort, electroterapie cu efect antialgic, miorelaxant, antiinflamator, stimularea metabolismului celular și a circulației arterio-venoase, efect în profunzime, efecte termice, masaj sedativ lombo-sacrat și ischiogambieni bilateral 

Folosirea ortezei lombare pentru susținerea peretelui abdominal anterior și corecția posturii coloanei lombare.

Evoluție favorabilă cu ameliorarea simptomatologiei algice, îmbunătățirea staticii și a dinamicii coloanei vertebrale, a mersului și a statusului funcțional.

Prognostic ad vitam: bun datorită lipsei comorbidităților și a evoluției favorabile în perioada postacută prognostic ad funcționam: satisfacător având în vedere evoluția favorabilă a statusului funcțional și a mersului, dar rămâne neclar sub amprenta compliantei și a statusului psiho-emoțional. Prognostic ad laborem: favorabil, pacientul recuperandu-și funcțiile necesare pentru reintegrare socio-profesională (muncă la birou) 

Particulatitatea cazului

Pacient cu antecedente de ulcer gastro-duodenal operat, hernie de perete abdominal, stenoză de canal lombar cu listezis L4, cu lombalgie cronică și parestezii plantare bilateral prezintă acutizări a simptomatologiei algice la acest nivel, fiind uneori diferit caracterul durerii și a traseului de iradiere.

Pacientul prestează muncă de birou, stătutul pe scaun timp întelungat agravează durerile cronice la nivelul coloanei lombare, timpul prelungit în fața calculatorului predispune la apariția simptomatologiei algice la nivelul coloanei cervical.

Cascada de evenimente survenite în timp a dus la patologia degenerativă a coloanei lombare cu apariția de modificări de stenoză de canal vertebral lombar, hernie de disc supraetajate, listezis.

Evoluția în timp a fost grevată și de necomplianța pacientului la tratament, nerespectarea indicației medicale și de sindromul psihoemoțional prezent. Având o deteriorare neurologică lentă abordarea terapiei conservatoare este cea mai indicată.

Recomandări:

• Regim igieno-dietetic hipolipidic, hiposodat, hidratare orală 2 L/zi. 

• Evită stresul fizic și psiho-emoțional, expunerea la temperaturi extreme

Tratament medicamentos: 

Pentoxi Retard 400 mg 1-0-1, Omez 20 mg 1-0-0, Rimalaya Forte 1-0-0, Liv 52 1-0-0, Prozac 20 mg 1-0-0 timp 2 luni, Mydocalm 150 mg 1-0-1 10 zile, Noliprel ARG forte 1-0-1 cu controlul TA.

Reevaluare cardiologică după 6 luni.

Va folosi orteză lombară pentru susținerea peretelui abdominal anterior și corecția posturii coloanei lombare.

Continuă programul KT învățat în clinică, respectă ”școala spatelui”

Tratament balnear : Calimănești-Căciulata, Felix, Herculane, Eforie Nord

# Meeting report: TERAPIA MIOFASCIALĂ ÎN PATOLOGIA POSTTRAUMATICĂ A MEMBRULUI INFERIOR

**Authors:** Meiu Lili, Nica Adriana, Mitoiu Brîndușa, Nartea Roxana, Vasilescu Toma, Constantinovici Mariana

**Affiliations:** * I.N.R.M.F.B. – Clinica Recuperare Medicina Fizică și Balneologie (III)

Sechelele post-traumatice ale membrului inferior afectează un număr foarte mare de pacienți care se adresează medicului de recuperare și pot determina pe termen mediu și lung dizabilitate în diferite grade.Un procent crescut din pacienții acestei categorii de patologii îl reprezintă persoanele tinere și active din punct de vedere profesional, de aici și nevoia de a găsi metodele de tratament cele mai eficiente și care să permită reluarea activității cât mai curând posibil.

Cele mai frecvente sechele traumatice la nivelul membrului inferior, implică genunchiul și glezna (fiind afectați atât persoanele obișnuite cât și sportivii). Intr-un studiu epidemiologic publicat în 2012, s-a constatat că un număr de 6,6 milioane de persoane au suferit un traumatism la nivelul genunchiului în Statele Unite (între 1999-2008) iar un studiu din 2010 a arătat că peste 3 milioane de persoane au suferit o entorsă de gleznă (2002-2006).

Programul complex de recuperare este alcătuit din mai multe segmente, care se adaptează pacientului dupa evaluarea clinico-funcțională efectuată de către medicul specialist de recuperare.

Dintre acestea se poate utiliza terapia miofascială-modalitate de tratament prin aplicarea de tehnici de terapie manuală cu acțiune asupra fasciei. Fascia reprezintă o rețea suplă, elastică de țesut conjunctiv, care îmbracă masele musculo-scheletale. Mulți pacienți ajung să beneficieze de terapia miofascială după pierderea flexibilității sau ca urmare a unei leziuni la nivelul oricărei zone care conține țesut moale.

Terapia miofascială utilizează 3 tehnici principale, care se pot combina în funcție de zona tratată (IASTM-Instrument Assisted Soft Tissue Mobilization,Trigger stick-instrument pentru punctele trigger,“Myofascial release”-terapie manuală asupra fasciei. Ședintele de tratament variază între 30-50 minute, asociind tehnici de respirație și relaxare efectuându-se la interval de 3-5 zile.

Din experiența Clinicii 3 în utilizarea acestei tehnici, am constatat control bun al sindromului dureros, cu sau fără asocierea celorlalte metode fizicale, scurtarea perioadei de tratament și reducerea polipragmaziei. De menționat că tratamentul a fost acceptat și tolerant de către pacienți.

In perspectivă urmărim evaluarea efectelor pe termen mediu și lung a terapiei miofasciale în ameliorarea suferințelor posttraumatice atât la nivelul membrului inferior cât și membrului superior și patologiei vertebrale.

# Meeting report: LOCUL ȘI ROLUL I.N.R.M.F.B. ÎN BALNEOLOGIA ROMÂNEASCĂ

**Authors:** Horia Lăzărescu, Lucian Mirescu

**Affiliations:** * I.N.R.M.F.B. – Departament Cercetare - Laborator de Studii Hidrogeologice Balneotehnice și Balneoclimatice a Factorilor Terapeutici Naturali

Lucrarea încearcă să selecteze scopurile principale ale existenței Institutului, ținând cont de cerințele societății și tendințele moderne în sănătate, prin promovarea principiilor ce privesc: 

• efectele curative recunoscute medical, probate științific și utilizate în tratament ale factorilor terapeutici naturali;

• proprietățile diferite ale factorilor terapeutici naturali;

• mecanismele acțiunii curei balneare;

• un anumit stil de viață, comportamente și medii favorabile pentru sănătate.

Sunt definite misiunile și funcțiile cheie ale institutului, cât și metodele de intervenție pe care și le propune, aducând ca exemplu câteva dintre obiectivele pe care I.N.R.M.F.B. și le-a propus pe termen apropiat și mediu.

# Meeting report: RECUPERAREA MEDICALĂ LA UN SECHELAR POST ACCIDENT VASCULAR CEREBRAL CU CARDIOMIOPATIE DILATATIVE ȘI FEVS 35%

**Authors:** Cătălina Miu, Miruna Săndulescu, Sebastian Diaconescu

**Affiliations:** * I.N.R.M.F.B. – Clinica Recuperare Medicală-Neurologie (IV)

Dintre toate afecțiunile neurologice ale adultului, cele cerebrovasculare se situează pe primul loc ca frecvență și importanță. Accidentul vascular cerebral este o afecțiune comună, dar severă, caracterizată de pierderea bruscă a funcțiilor neurologice prin întreruperea fluxului sangvin la nivel cerebral. Este cea mai importantă cauză de dizabilitate fizică din țările dezvoltate, având importante implicații individuale, dar și asupra familiei și societății.

In funcție de mecanismele de producere, accidentele vasculare cerebrale pot fi clasificate în accidente vasculare ischemice (85%) sau hemoragice (15%). Factorii de risc sunt reprezentați de vârstă (riscul de producere a unui accident vascular cerebral crește odată cu vârstă), sexul (bărbații sunt mai frecvent afectați, însă după vârsta de 75 de ani femeile devin cele la care se întâlnește mai frecvent accidentul vascular), hipertensiunea arterială, diabetul zaharat, diverse afecțiuni ale inimii (afecțiuni ale valvelor cardiace, cardiomiopatiile, endocarditele, fibrilația atrială), existent unui accident vascular cerebral în antecedente sau în familie, obezitatea, fumatul, diverse medicamente (anticoncepționale orale, anticoagulante, corticosteroizi), consumul excesiv de alcool, consumul de droguri. 

Majoritatea dizabilităților secundare accidentului vascular cerebral pot fi recuperate în câteva luni, însă altele pot să persiste pentru restul vieții. Reabilitarea trebuie începută cât mai repede, deoarece există o șansă mai mare de recuperare în stadiul precoce. Dizabiliățile rămân permanente și se accentuează odată cu trecerea timpului, de aceea instituirea unui program de recuperare cât mai curând posibil este esențial.

Metodele de reabilitare după un accident vascular cerebral diferă de la un pacient la altul, însă au același scop: dobândirea unui status funcțional care să ofere independență și ajutor minim din partea altor persoane, acomodarea fizică și psihică a pacientului cu schimbările determinate de accidentul vascular, precum și integrarea corespunzătoare în familie și comunitate.

Pacientul G.D., în vârstă de 57 de ani, de sex masculin, pensionar, domiciliat în mediul rural, a fost internat în Clinica IV a Institutului Național de Recuperare, Medicină Fizică și Balneoclimatologie București în perioda (....), pentru reevaluare clinico-funcțională și continuarea programului de recuperare, prezentând deficit motor la nivelul hemicorpului drept (predominant brahial), afazie mixtă (predominant expresivă), tulburare moderată de deglutiție pentru lichide, disfuncție moderată de locomoție și autoîngrijire, instalate în urma unui accident vascular cerebral ischemic cu transformare hemoragică în teritoriul arterei cerebrale medii stângi în data de 08.04.2016.

Din antecedentele personale patologice ale pacientului se reține: accident vascular cerebral vechi în teritoriul arterei cerebrale medii drepte (2007), infarct miocardic anteroseptal (2009), anevrism apical fără tromboză intraapicală, cardiopatie ischemică cu diskinezie septo-apicală cu FEVS conservată, hipertensiune arterială stadiul II grup de risc adițional înalt, diabet zaharat II, hipertrigliceridemie, ateromatoză cervico-cerebrală moderată, spondilită anchilozantă (1993), coxartroză secundară bilaterală, polip esofagian ulcerat (aprilie 2016), criză convulsivă generalizată nedocumentată (iulie 2016).

Examenul obiectiv la internare: pacient conștient, afazie mixtă, predominant expresivă, rare raluri bronșice diseminate în ambele câmpuri pulmonare, tuse ineficientă, TA= 130/70 mmHg, AV=68 b/min, aria matității cardiace marită lateral stânga, suflu discret stenoza aortică, artere periferice pulsatile, abdomen nedureros spontan și la palpare, tranzit intestinal prezent, micțiuni fiziologice.

Examenul aparatului neuromusculo-artro-kinetic a relevat: frustă pareză facială dreapta, deficit motor paretic la nivelul hemicorpului drept, predominant brahial, spasticitate grad I flexori cot, pumn și degete, grad II extensori genunchi, reflexe osteotendinoase vii, schiță clonus plantar, Babinski prezent, control motor moderat proximo-distal la nivelul membrului superior drept, prezent proximo-intermediar, moderat distal la nivelul membrului inferior drept, sensibilitate și probele de coordonare nu au putut fi testate în contextul afaziei, limitarea globală a mobilității pasivo-active a articulației coxo-femurale drepte.

Din punct de vedere funcțional, opoziția policelui, prehensiunea și pensele au fost posibile, dar realizate cu dificultate, efectua singur mobilitatea în pat, transferul din clinostatism în poziția șezut scurtat la marginea patului și din șezut în ortostatism au fost realizate independent, menținea ortostatismul fără ajutor și se deplasa pe distanțe scurte și medii cu sprijin în cârjă canadiană, cu schema modificată: mers spastic, cosit, flexie insuficientă de genunchi, atac digitigrad, risc moderat de dezechilibru și cădere.

Pe parcursul internării curbele fiziologice (puls, TA, diureza, temperatura) au fost în limite normale. Pentru a evalua rezerva generală vitală a pacientului au fost cerute următoarele investigații paraclinice:

• HLG: în limite normale

• Biochimie: normale, cu excepția creatininei (2,05 mg/dl), ureei (137,2 mg/dl), acidului uric (8,5 mg/dl)

• Profil lipidic: trigliceride (164 mg/dl)

• Probe inflamatorii: negative

• Sumar urină: proteinurie 100 mg/dl, leucocite relativ frecvente

• EKG: RS, Ax Qrs +60 grade, necroză anterioară întinsă, bigeminism ventricular, ESV monomorfe, monofocale cu aspect BRD, fără tulburari de repolarizare.

Având în vedere modificările apărute pe EKG-ul de la internare la un pacient cu un bogat istoric cardiovascular, s-a decis montarea unui Holter EKG pe 24 de ore și solicitarea unui consult interdisciplinar cardiologic, din care a rezultat următorul diagnostic: Cardiomiopatie ischemică dilatativă, ICC III NYHA FEVS =35-40%, BCI, ESV cu tendință la sistematizare, HTA II, BCR III;

De asemenea, s-a considerat necesară și o reevaluare neurologică ținând cont de istoricul recent de criză convulsivă generalizată la un pacient cu doua accidente vasculare cerebrale în antecedente. Astfel, la diagnosticele deja cunoscute s-a adaugat și cel de epilepsie vasculară, motiv pentru care a fost considerat necesar instituirea tratmentului cu anticonvulsivant: Levetiracetam 500 mg 1+1/2 cp/zi.

La această internare, tratamentul realizat a avut următoarele obiective:

• Terapia afecțiunilor cardiovasculare asociate pentru a crește rezerva funcțională a pacientului

• Prevenția secundară a AVC-ului

• Creșterea nivelului de independență motorie și reintegrarea socio-familială prin:

- Creșterea controlului motor la nivelul hemicorpului drept

- Antrenarea abilității necesare traiului zilnic (ADL-uri)

- Antrenamentul mobilității în pat și a transferurilor

- Antrenamentul mersului și corectarea schemei de mers

- Menținerea mobilității articulare

- Ameliorarea tulburărilor de deglutiție 

- Ameliorarea tulburărilor de comunicare

Deși tratamentul de recuperare a fost unul complex, pacientul a fost staționar din punct de vedere funcțional la această internare, evoluția fiind grevată de patologiile coexistente, dar și de o participare selectivă la programul de reabilitare. In ciuda acestui lucru, evoluția globală, de la ultimul accident vascular până în prezent, este favorabilă – pacientul prezintă un grad ridicat de independență. In schimb prognosticul vital rămâne rezervat, existând riscul recidivei accidentului vascular cerebral datorită numeroșilor factori de risc asociați: hipertensiune arterială, diabet zaharat, ateromatoză cervicocerebrală moderată, cardiopatie ischemică, hipetrigliceridemie, accident vascular cerebral vechi în teritoriul arterei cerebrale medii drepte, infarct miocardic anteroseptal sechelar.

# Meeting report: CERINȚE ÎN ATESTAREA STAȚIUNILOR BALNEOCLIMATICE

**Authors:** Mirescu Lucian, Truică Ion, Slavu Ben Alexandru, Pârvulescu Teodor

**Affiliations:** * I.N.R.M.F.B. – Departament Cercetare - Laborator de Studii Hidrogeologice Balneotehnice și Balneoclimatice a Factorilor Terapeutici Naturali

Lucrarea se referă la turismul balnear românesc, la caracteristicile sale particulare și mai ales la tradiția istorică a tratamentului balnear în România.

Turismul balnear poate avea un impact indiscutabil, în termeni economici, în plan regional și este foarte legat de politica națională de apărare a sănătății populației.

Lucrarea reprezintă un studiu clasic asupra unor capitole bine cunoscute, precum:

- dezvoltarea şi modernizarea infrastructurii de transport; 

- protejarea şi îmbunătăţirea calităţii mediului; 

- dezvoltarea resurselor umane, promovarea ocupării şi a incluziunii sociale şi întărirea capacităţii administrative; 

Se încearcă găsirea și explicarea în vederea aplicării a principiilor generale cu “Obiective” de aplicat în vederea obținerii unor rezultate precum:

- creșterea eficienței și calității actului medical

- creșterea satisfacției pacienților 

- creșterea satisfacției personalului medical

Toate acestea reprezintă modalități de reabilitare a sectorului balnear românesc și o pledoarie pentru redescoperirea unui domeniu, turismul balnear în care țara noastră era foarte bine cunoscută și apreciată.

# Meeting report: MANAGEMENTUL RISCULUI DE CĂDERE LA VÂRSTNIC

**Authors:** Gilda Mologhianu, Marius Ivașcu, Valentin Șerban

**Affiliations:** * I.N.R.M.F.B. – Clinica Recuperare Medicină Fizică și Balneologie (III)

Căderile, prin consecințele lor, reprezintă o importantă cauză de dizabilitate la populația vârstnică. Ele sunt datorate tulburărilor de echilibru, tulburărilor de percepție și scăderii forței musculare, sarcopeniei vârstnicului. Căderile la vârstnici sunt determinate rareori de o cauză unică, iar o prevenire eficientă necesită o apreciere comprehensivă a deficițelor intrinseci ale pacientului (de obicei afecțiuni caracteristice vârstei înaintate, consumului de medicamente), dar și a obstacolelor din mediu.

Complicațiile redutabile ale căderilor sunt afectările de la nivelul aparatului locomotor, îndeosebi fracturile. Una din patru persoane care cade suferă leziuni serioase. 5% dintre căderi determină fracturi și într-o proporție egală provoacă afectare a țesutului moale. Căderile sunt a șasea cauză principală de deces la vârstnici. Teama de cădere va duce la pierderea independenței pacientului, la apariția sindromelor de decondiționare ce vor accentua și mai mult dizabilitatea persoanei în cauză.

Ne-am propus să dezvoltăm acest subiect cu implicații sociale și economice importante în condițiile creșterii speranței de viață și deci a segmentului de populație vârstnică și să expunem câteva principii de management în vederea creșterii calității vieții persoanelor cu risc de cădere.

# Meeting report: POLINEUROPATIA SENZITIV-MOTORIE PREZENTARE DE CAZ

**Authors:** Naglabeala Ovidiu, Mihaela Purcaru, Daniela Poenaru, Horia Lăzărescu

**Affiliations:** * I.N.R.M.F.B. – Clinica Recuperare Medicală-Neurologie (IV)

Introducere. Afecțiunile sistemului nervos periferic sunt frecvente dar uneori greu de diagnosticat. Neuropatiile periferice au un impact major în calitatea vieții pacienților și a independenței funcționale. In plus pacienții cu neuropatie au un risc de 20 de ori mai mare de a cădea comparativ cu pacienții fără neuropatie.

Rezumat. In continuare prezentăm cazul unui pacient în vârstă de 87 ani fără antecedente personale patologice semnificative care se prezinta în clinică pentru deficit motor paraparetic și disfuncție severă de locomoție și autoîngrijire. Debutul afecțiunii are loc, cu o lună înainte de internare în clinică, brusc cu deficit motor paraparetic soldat cu cădere de la propriul nivel. Ulterior se pune diagnosticul de polineuropatie senzitiv-motorie axonal demielinizantă. Evoluția pe parcursul internării a fost favorabilă cu inițierea ortostatismului și a mersului cu ajutorul cadrului. Din punct de vedere al independenței funcționale pacientul se externează cu scor FIM 5 având nevoie de însoțitor permanent pentru supraveghere.

Concluzie. O polineuropatie debutată brusc cu paraplegie, la o persoană vârstnică, independentă funcțional, până în acel moment, cu evoluție relativ bună a deficitului motor în cursul programului de recuperare, dar care nu-i restituie independență funcțională.

# Meeting report: PARAPAREZA SPASTICĂ INCOMPLETĂ PRIN DUBLU MECANISM COMPRESIV

**Authors:** Mihaela Purcaru, Ovidiu Naglabeala, Daniela Poenaru, Horia Lăzărescu

**Affiliations:** * I.N.R.M.F.B. – Clinica Recuperare Medicală-Neurologie (IV)

Pacienta LV, 63 ani, din mediu urban, cunoscută cu HTAE std I controlată medicamentos, boală cardiacă ischemică, dislipidemie cu hipercolesterolemie, ciroză hepatică toxic-nutrițională, gonartroză genunchi drept, care instalează în anul 2013 o parapareză spastică incompletă prin dublu mecanism compresiv în urma unui abces epidural T5-T6 (operat în martie 2013) și a unei osteomielite la nivelul T5 (operată în aprilie 2013, cu montarea unui fixator posterior din titan la nivelul T3-T6), se internează în clinica noastră pentru deficit motor de tip paraparetic, disfuncție locomotorie și de autoîngrijire moderat-severă , precum și, dureri în regiunea genunchiului drept.

Clinic, pacienta are o stare generală bună , este cooperantă, OTS și fără semene meningeale, echilibrată pulmonar și cardiovascular, fără semen clinice patologice pe aparatele digestiv și uro-genital.

In cadrul examenului NMAK, patologic se remarcă, la nivelul membrelor inferioare, deficit de tip paretic, control motor proximo-intermedio-distal present (dreptul<stangul), spastcitate pe cvadriceps și adductori de gr. I-II pe dreapta, gr. I pe stânga, ROT vii (drepta>stânga), Babinski prezent bilateral, retractura bilaterala de tendon Achilean. Din punct de vedere functional realizeaza mobilitatea în pat și transferurile cu mare dificultate (cu folosirea unor dispozitive improvizate pentru tracțiune cu membrele superioare), menține poziția șezut la marginea patului timp limitat de sindromul algic lombar, ambulația se realizează cu sprijin în cadru pe distanțe scurte, pașii fiind nesiguri, mici și inegali (stângul<dreptul).

Evoluția pe parcursul internării este favorabilă, cu o îmbunătățire considerabilă a mobilității în pat, transferurilor și a mersului, astfel că, la externare ambulația se realizează cu sprijin în două cârje canadiene.

# Meeting report: SINDROMUL DUREROS LOMBO-SACRO-FESIER ÎN SARCINĂ

**Authors:** Roxana Nartea, Nica Sarah Adriana

**Affiliations:** * I.N.R.M.F.B. – Clinica Recuperare Medicină Fizică și Balneologie (III)

O parte dintre afecțiunile musculoscheletale la gravide apar datorită modificărilor de postură, care pe de o parte schimbă centrul gravitațional, iar pe de altă parte obligă femeia la poziții compensatorii pentru a-și menține echilibrul. Creșterea în volum a abdomenului și a glandelor mamare sunt modificările cele mai importante care obligă gravida la adoptarea unor modificări posturale, cele mai evidente fiind:

1. La nivelul coloanei și bazinului: anteflexia gâtului și extensia ușoară a capului, hipercifoza, hiperlordoza, anteversia bazinului

2. La nivelul membrelor superioare: umerii cazuți

3. La nivelul membrelor inferioare: coapsele în abducție și rotație externă, genunchii în hiper extensie, picioarele în ușoară pronație și bolta plantară prabușită

 Modificările musculare sunt tipice și constau în contracția reflexă a flexorilor șoldului și a mușchilor regiunii lombare și pectorale. Acest lucru poate provoca apariția durerii datorită suprasolicitării.

Van Barten E, Pool J, Pool-Goudzwaard A au publicat în Journal of Orthopaedic & Sports Physical Therapy in 2014 o analiză sistematică a literaturii de specialitate (PubMed, Pedro, Scopus si CINAHL) din ianuarie 1992 până în noiembrie 2013 privind utilitatea exercițiului fizic pentru tratamentul durerii lombare joase în timpul sarcinii. Au fost incluse un total de 22 de articole (toate fiind studii randomizate și controlate) de raportare a 22 de studii independente. Au fost colectate date privind 4 tipuri de intervenții: un program complex de recuperare (7 studii, n= 1202), proframul kinetic (9 studii, n= 2149), terapie manuală (5 studii, n=360), stijin material (1 studiu, n=115). Concluziile acestei analize sistematice au ca programul de recuperare complex ce include kinetoterapie, terapie manuală, educația pacientului are un efect pozitiv asupra durerii, diminuând semnificativ numărul de zile de concediu medical.

# Meeting report: COMPLICAŢII CARDIOVASCULARE LA PACIENŢII CU TRAUMATISME VERTEBRO-MEDULARE: EVALUAREA FACTORILOR DE RISC

**Authors:** Ioana-Simona Neagoie, Liliana Cioc, Renee Popovici, Delia Cinteză, Horia Lăzărescu

**Affiliations:** * I.N.R.M.F.B. – Clinica Recuperare Medicală-Neurologie (IV)

Introducere. Studii recente au raportat creşterea mortalitǎţii de cauze cardiace la pacienţii cu leziuni medulare cronice. Deşi factorii de risc sunt cei descrişi la populaţia generalǎ, se pare cǎ prevalenţa lor este mai ridicatǎ în rândul pacienţilor cu traumatisme vertebro-medulare. Printre condiţiile favorizante se numǎrǎ tulburarea metabolismului lipidelor şi carbohidraţilor, precum şi asocierea unui status proinflamator. De asemenea, disreflexia autonomǎ este implicatǎ prin modificarea valorilor tensionale, tulburǎrile de ritm cardiac şi scǎderea capacitǎţii de efort. 

Obiective. Identificarea şi prezentarea afecţiunilor cardiovasculare diagnosticate la pacienţii cu leziuni medulare cronice şi dependenţǎ functionalǎ, dar şi a mecanismelor etiopatogenice implicate.

Material şi metodǎ. La pacienţii cu leziuni medulare, imobilizarea prelungitǎ este asociatǎ cu modificarea metabolismului muscular, reducerea nivelului de HDL colesterol, obezitate şi creşterea rezistenţei la insulinǎ-factori care predispun la leziuni coronariene de tip ischemic. Din cauza întreruperii transmiterii nervoase şi a limitǎrii activitǎţii fizice, aceste evenimente coronariene pot sǎ treacǎ neobservate. Recent, s-a demonstrat imagistic (EBT-electron beam computerized tomography) existenţa unui grad mai ridicat de calcificǎri la nivelul arterelor coronare comparativ cu populaţia generalǎ de aceeaşi vârstǎ, rasǎ şi sex. Diagnosticarea şi tratarea factorilor de risc reprezintǎ o componentǎ importantǎ a programului terapeutic şi poate juca un rol important în reducerea incidenţei afecţiunilor cardiovasculare la acest grup de pacienţi.

Rezultate. Afecţiunile cardiovasculare reprezintǎ în prezent principala cauzǎ de morbiditate şi mortalitate în rândul populaţiei cu leziuni medulare, fiind incriminaţi atât factori de risc din populaţia generalǎ, cât şi anumite condiţii favorizante particulare, dintre care imobilizarea şi disreflexia autonomǎ par sǎ joace cel mai important rol.

Concluzii. Cunoaşterea factorilor de risc pentru afecţiunile cardiovasculare în rândul populaţiei cu leziuni medulare este importantǎ pentru adoptarea celor mai potrivite strategii terapeutice. În prezent, sunt puţine trialuri clinice care fac referire la condiţiile clinice şi de viaţǎ care predispun la aceste tulburǎri, fiind necesare studii suplimentare pentru o mai bunǎ înţelegere a mecanismelor implicate şi a mǎsurilor terapeutice eficiente.

# Meeting report: SARCOPENIA ŞI FRAGILITATEA FIZICǍ LA PACIENTUL VÂRSTNIC-METODE DE EVALUARE ŞI TRATAMENT

**Authors:** Ioana-Simona Neagoie, Liliana Cioc, Codruța Soare, Mirabela Dragomir, Delia Cinteză, Horia Lăzărescu

**Affiliations:** * I.N.R.M.F.B. – Clinica Recuperare Medicală-Neurologie (IV)

Introducere. Noţiunile şi definiţiile sarcopeniei şi fragilitǎţii au fost în mare mǎsurǎ revizuite de-a lungul timpului, iar în prezent se considerǎ cǎ ele pot fi combinate într-o definiţie unicǎ. Dacǎ sarcopenia este definitǎ prin scǎderea progresivǎ şi generalizatǎ a masei şi a forţei musculare scheletice, fragilitatea reprezintǎ o depreciere multisistemicǎ asociatǎ cu creşterea vulnerabilitǎţii la factori de stres şi a predispoziţiei la diferite afecţiuni cronice, fiind în mare parte mediatǎ de sarcopenie. 

Obiective. Prezentarea mǎsurilor de evaluare clinicǎ şi funcţionalǎ şi a metodelor de tratament la pacienţii vârstnici, fragili, cu diferite grade de dizabilitate.

Material şi metodă. Pentru evaluarea clinicǎ a celor douǎ sindroame se folosesc în prezent diferite scale , precum algoritmul elaborat de Grupul European de Lucru al Sarcopeniei (European Working Group on Sarcopenia in Older People) sau scala FRAIL. Mǎsurile terapeutice sunt complexe: de la evitarea medicamentelor care pot sǎ inducǎ pierderea masei musculare şi diete care cresc sinteza proteinelor, la programe de kinetoterapie cu accent pe antrenamentul neuromuscular , antrenamentul echilibrului, prevenirea cǎderilor şi fracturilor.

Rezultate. Studii observaţionale şi o serie de trialuri clinice randomizate au arǎtat efectele benefice ale exerciţiului fizic asupra nivelului funcţional şi al gradului de independenţǎ la pacienţi vârstnici cu diferite grade de dizabilitate.

Concluzii. Recunoaşterea sarcopeniei ca şi componentǎ majorǎ a fragilitǎţii fizice sugereazǎ faptul cǎ mǎsurile terapeutice care vizeazǎ muşchiul scheletic pot avea efecte benefice asupra fragilitǎţii şi corelaţiilor sale clinice. În acest context, sunt necesare studii suplimentare care sǎ conducǎ la o mai bunǎ definire a celor douǎ entitǎţi şi recunoaştere a pacienţilor cu risc de sarcopenie şi fragilitate.

# Meeting report: ACTUALITAȚI ÎN RECUPERAREA FUNCȚIONALĂ A PACIENȚILOR CU SCLEROZĂ MULTIPLĂ

**Authors:** Florina Ojoga

**Affiliations:** * I.N.R.M.F.B. – Clinica Recuperare Medicină Fizică și Balneologie (III)

Scleroza multiplă este o patologie care debutează frecvent la adultul tânăr între 20-40 ani, raportul femei/bărbați fiind de 3/2, mai ales în regiunile temperate și reci.

Leziunile anatomice sunt reprezentate de plăci demielinizante multiple în substanță albă a axului cerebro-spinal, cu predilecție medulară, periventriculară și cerebeloasă.

Mecanismul etiopatogenic este probabil autoimun, cu anumiți factori declanșatori (virusuri, antigene proteice).

Tabloul clinic este variat, cu tulburări motorii (spasticitate, pareză, paraplegie), tulburări de echilibru și coordonare, tulburări de sensibilitate, sfincteriene și vizuale.

Recuperarea medicală are ca obiective păstrarea independenței funcționale în ceea ce privește locomoția, prevenirea atitudinilor vicioase, reducerea spasticității și menținerea funcției ADL-urilor și se realizează cu mijloace specifice de tratament: kinetoterapie, ergoterapie, tehnici de recuperare funcțională, sociala și profesională.

# Meeting report: RECUPERAREA AFAZIEI LA PACIENTUL CU A.V.C.

**Authors:** Olariu Teona

**Affiliations:** * I.N.R.M.F.B. – Clinica Recuperare Medicală-Neurologie (IV)

Accidentul vascular cerebral este a III cauza de mortalitate, după bolile cardio-vasculare și neoplazii. Este o patologie invalidantă cu semne și simtome ce variază în funcție de teritoriul afectat: deficitul motor contralateral leziunii; afazie, apraxie, dizartrie; hemianopsie, tulburări ale conștienței, nistagmus, vertij, ataxie.

Afazia este o tulburare caracterizată prin pierderea parțial sau totală a capacității de a percepe informația sau de a se exprima verbal sau în scris. Ea poate fi: motorize sau expresivă (Broca), senzorială sau receptive (Wernicke) și globală. Recuperarea completă, spontană a afaziei, după un AVC are un procent de 21-25% în primele 8-12 săptămâni, după care prognosticul de recuperare scade. Terapia logopedică trebuie începută în măsura în care pacientul este în măsură să colaboreze cu logopedul, la care se adaugă și suportul familial.

Importanța recuperării pacientului afazic, este dată de reintegrarea socio-profesională, familială și creșterea calității vieții individului. Reprezintă un domeniu complex de activitate.

# Meeting report: POLINEUROPATIA DEMIELINIZANTĂ INFLAMATORIE CRONICĂ - CAZ CLINIC

**Authors:** Pavel Ana-Maria, Diaconescu Sebastian, Horia Lăzărescu

**Affiliations:** * I.N.R.M.F.B. – Clinica Recuperare Medicală-Neurologie (IV)

 Polineuropatia demielinizantă inflamatorie cronică (PDIC) este o patologie dobîndită, rară, de origine necunoscută, prezumtiv imunologică care afectează sistemul nervos periferic. Este cauzată de deteriorarea învelișului protector al nervilor (mielina). Boala afectează orice vârstă, ambele sexe; Incidența este de 0,8 la 7.7/100.000. Clinic: disfuncție motorie și senzitivă, progresivă sau cu recăderi, care interesează cel puţin două membre, însoțită de hipo- sau areflexie. Pentru diagnosticarea PDIC se efectuează următoarele investigații paraclinice: puncție LCR (disociație albumnio-citologica), examen electrofiziologic EMG: blocuri de conducere multifocale, demielinizări segmentare, latențe distale prelungite, scăderea vitezelor de conducere.

Am examinat pacienta M.C., de sex feminin, în vârstă de 60 ani, care s-a prezentat în clinica noastră prin transfer de la spitalul Municipal pentru: deficit motor tetraparetic cu predominanța paraparezei, disfuncție locomotorie și de autoîngrijire moderată și durere cu caracter neuropat la nivelul membrelor inferioare, pentru evaluare clinico-funcțională și stabilirea unui program de recuperare adaptat cazului.

# Meeting report: SINDROMUL DE HEMINIGLIJENȚĂ SPAȚIALĂ

**Authors:** Pavel Ana-Maria, Diaconescu Sebastian

**Affiliations:** * I.N.R.M.F.B. – Clinica Recuperare Medicală-Neurologie (IV)

 Neglijarea spațială unilaterală este un complex, dar fascinant, deficit de atenție, care poate să apară după un accident vascular cerebral. Organizarea neuronală a orientării spațiale arată o dominanță a emisferei drepte. Emisfera dreaptă direcționează atenția spre întregul spațiu extrapersonal, în timp ce emisfera stângă direcționează atenția numai către hemispațiul drept contralateral. Lezarea unilaterală a emisferei stângi nu determină neglijare contralezională, din moment ce mecanismele atenționale ipsiversive ale emisferei drepte pot compensa pierderea funcțiilor emisferei stângi de direcționare contralaterală a atenției. Totuși, lezarea unilaterală a emisferei drepte determină neglijare hemispațială stânga contralezională severă, deoarece emisfera stângă neafectată nu conține mecanisme atenționale ipsiversive. In consecință, neglijarea contralezională este mult mai frecventă, severă și de durată după lezarea emisferei drepte decât după lezarea celei stângi. Neglijarea severă pentru hemispațiul drept este rară, chiar și la stângacii cu leziuni în emisfera stângă.

Pacienții cu neglijare severă pot fi incapabili să se îmbrace, să se radă sau să-și îngrijească partea stangă a corpului; nu pot mânca ceea ce este așezat în partea stângă a farfuriei și nu pot citi jumătatea stângă a frazelor.

# Meeting report: RECUPERAREA MEDICALĂ ÎN FRACTURILE DISTALE DE RADIUS

**Authors:** Viorel Petcu, Ana Maria Pavel, Elena Stănciulescu, Sebastian Diaconescu, Horia Lăzărescu

**Affiliations:** * I.N.R.M.F.B. – Clinica Recuperare Medicală-Neurologie (IV)

 Fracturile distale de radius sunt printre cele mai frecvent întâlnite patologii în practica ortopedică adultă și reprezintă 1/6 dintre fracturile tratate în departamentele de urgență. Sunt întâlnite mai frecvent la adulții tineri și persoanele cu vârsta peste 65 de ani cu o incidență variabilă între aceste grupuri iar cele mai multe apar ca urmare a căderii pe mâna întinsă cu încheietura în extensie. La adulții tineri, fracturile apar în urma traumatismelor cu energie mare de impact, care implică o combinație de îndoire, compresie, impact și forfecare iar la adulții în vârstă, ca urmare a traumatismelor de energie joasă, cum ar fi căderea de la propriul nivel, de obicei pe os osteoporotic. Tipul de fractură rezultat este de asemenea caracteristic grupei de vârstă, astfel la pacienții tineri predomină fracturile cominutive metafizare, care pot implica injurii masive ale suprafeței articulare și a cartilajului triunghiular iar la pacienții în vârstă se întâlnesc mai frecvent fracturile extraarticulare, angulate dorsal sau cu deplasare a fragmentelor. In general, fracturile sunt: intraarticulare (cu sau fără deplasare), extraarticulare (cu sau fără deplasare), fracturile deschise și fracturile cominutive. Unele dintre cele mai frecvente tipuri de fractură este fractura Colles, prin protruzia posterioară a extremității radiale distal de fractură, fractura Smith (Colles inversată), fractura Barton (volară sau dorsală, totdeauna intraarticulară și implică subluxatie carpiană), fractura Hutchinson (a șoferului- comprezia axială a scafoidului în fatela radială), fractura Galleazi (sau Piemont, jontiunea 1/3 cu 2/3 distale a radiusului, asociată cu subluxatia joncțiunii radioulnare distale) și fracturile radiale distale pediatrice.

In ceea ce privește tratamentul, deși chirurgia este luată în considerare pentru fracturile cu deplasare sau instabile, opțiunile terapeutice optime depind de câțiva factori, scopul fiind de a asigura o funcționalitate cât mai apropriată de cea inițială. Pentru pacienții în vârstă, mai puțin activi, sau adulții cu necesități fizice reduse, reducerea închisă sau aparatul ghipsat sunt întotdeauna preferate. O analiză sistematică a studiilor existente privind managementul fracturilor radiale distale la pacienți peste 60 de ani, utilizând 5 tehnici comune de fixare, publicată în The Journal of Hand Surgery în 2011, a sugerat că, în pofida rezultatelor radiografice mai puțin favorabile asociate cu imobilizarea ghipsată, rezultatele funcționale nu au fost diferite de cele obținute în urma tratamentului chirurgical la pacienții cu vârstă peste 60 de ani.

Programul de recuperare pentru membrul superior post traumatic urmărește recuperarea funcțiilor normale ale mâinii și se adaptează dezvoltările patologiei individuale, cu mobilizare precoce a degetolor activă asistată sau activă, pentru menținerea lanțurilor de mișcare și a penselor și se asociază proceduri fizicale cu efect analgezic, vasculotrop, spasmolitic și simpaticolitic. 

O analiză comparativă a datelor disponibile din 26 de studii clinice randomizate, publicate în baza de data Cochrane, arată că acestea nu sunt sufciente pentru a stabili eficiența comparativă a diverselor metode de reabilitare utilizate: fizioterapie de rutină, terapie ocupațională, mobilizare precoce a mâinii.

# Meeting report: TRATAMENTUL DE RECUPERARE ÎN BOALA ARTROZICĂ LA PACIENTUL VÂRSTNIC

**Authors:** Irina Petrușcă, Delia Cineză, Victorița Suciu, Sebastian Diaconescu, Stelian Sorica, Lavinia Sodringa

**Affiliations:** * I.N.R.M.F.B. – Clinica Recuperare Medicală-Neurologie (IV)

Introducere: Boala artrozică afectează peste 20 mil adulți în SUA. Peste jumătate din persoanele peste 65 ani prezintă boala artrozică, principala cauză de dizabilitate la această grupă de vârstă cu un impact economic de peste 60bil $/an.

Obiective: Datorită lipsei existenței unui tratament curativ, obiectivele principale sunt de a minimaliza efectele și consecințele bolii artrozice.

Material și metoda: Inițial considerată ca o afecțiune datorată îmbătrânirii sau uzurii cartilajului, astăzi artroza este considerată o afecțiune cu polietiologie și polipatogenie. Modificarile patologice din cadrul artrozei sunt consecința unei largi varietăți de factori etiologici ce duc în final la alterarea funcționalității mecanice articulare și afectarea structurală a cartilajului articular. Tratamentul este unul complex, adaptat având ca obiectiv ameliorarea durerii și creșterea statusului funcțional, crescând în final calitatea vieții pacientului.

Rezultate: Studii realizate de AHRQ (Agency for Health care Research and Quality) indică faptul ca tratamentul medicamentos, terapia ocupațională, tratamentul chirurgical pot reduce durerea, menține/îmbunătăți mobilitatea articulară și pot limita dizabilitatea funcțională.

Concluzii: este nevoie de un plan de tratament adaptat fiecărui pacient în parte în care pacientul devine un “partener” . AHRQ a demonstrat cum persoanele vârstnice cu boala artrozică devin mai active și mai responsabile, reduc costurile pentru îngrijiri medicale și își îmbunătățesc calitatea vieții după un tratament adaptat/personalizat.

# Meeting report: ASPECTE ECOGRAFICE ALE UMĂRULUI DUREROS LA PACIENTUL ECOGRAFIC

**Authors:** Daniela Poenaru

**Affiliations:** * I.N.R.M.F.B. – Clinica Recuperare Medicală-Neurologie (IV)

Obiective: identificarea modificărilor anatomice asociate umărului dureros la pacientul cu accident vascular cerebral cronic.

Metode: studiu longitudinal pe 16 pacienți cu accident vascular cerebral vechi (18-24 luni), cu umăr dureros. S-au urmărit parametri ecografici și evaluarea funcțională pe scale specifice.

Rezultate: mărirea distanței dintre arcul acromioclavicular și tuberculul mare se corelează negativ cu funcționalitatea membrului superior.

# Meeting report: PROTOCOL DE TRATAMENT ÎN ENTORSELE DE GENUNCHI

**Authors:** Daniela Poenaru

**Affiliations:** * I.N.R.M.F.B. – Clinica Recuperare Medicală-Neurologie (IV)

Diagnosticul entorselor de genunchi se bazează din ce în ce mai mult pe examenul ecografic. Acesta permite stabilirea cu precizie a tipului de leziune, a integrității structurilor genunchiului și este o etapă diagnostică importantă.

Entorsele de genunchi beneficiază de tratament de recuperare, cu certitudine pentru gradele 1 și 2, si din ce în ce mai mult pentru gradul 3, în detrimentul tratamentului chirurgical. 

Etapele programului de recuperare trebuie identificate și urmărite cu conștiinciozitate iar evoluția leziunilor se poate face ecografic.

# Meeting report: CREȘTEREA NIVELULUI FUNCȚIONAL ȘI DE INDEPENDENȚĂ LA PACIENȚII NEUROLOGICI PRIN INTERMEDIUL F.E.S.

**Authors:** Renee Popovici, Simona Neagoie, Liliana Cioc, Irina Petrușcă, Mirabela Dragomir

**Affiliations:** * I.N.R.M.F.B. – Clinica Recuperare Medicală-Neurologie (IV)

Introducere: Stimularea electrică funcțională (FES) este o metodă terapeutică ce folosește curentul electric de intensitate joasă (rectangular) pentru a redobândi sau îmbunătăți funcțiile pierdute secundar unei afecțiuni neurologice.

FES nu este un tratament ci doar un dispozitiv ajutător prin care pacientul își crește nivelul de independență, doar pe parcursul folosirii acestui sistem. Prin stimularea electrică funcțională se urmarește contracția musculară și suplinirea unei funcții deficitare/pierdute.

Este aplicată pe nervii periferici care controleză grupe musculare sau mușchi specifici normal inervați (fiind ineficientă pe musculatura denervată).

Obiective: Reactualizarea și trecerea în revistă a indicațiilor și beneficiilor utilizării FES în managementul teraputic al patologiilor neurologice, cu evidențierea contribuirii la creșterea funcționalității și, implicit, a calitatii vietii pacientilor.

 Materiale si metode: Materiale sunt selectate din literatura de specialitate, internațională. Metodele de tratament se referă la dispozitive adaptate/concepute pentu stimularea electrică funcțională și parametrii stabiliți pentru ele; aplicații, beneficii, specificații tehnice, avantaje, dezavantaje.

Sunt stabilite recomandările pentru utilitatea clinică, care sunt factorii, parametrii relevanți care asigură efectele terapeutice și nu în ultimul rând, un factor economic important, analiza cost-beneficii.

Rezultate și concluzii: FES reprezintă una dintre metodele prin care pacientul cu afectare a SNC își poate crește nivelul de funcționalitate, cu îmbunătățirea calității vieții. Nu reprezintă un procedeu curativ, doar adjuvant. Dispozitivele concepute sunt rezultatul colaborării îndelungate dintre ingineri, clinicieni și bineințeles, pacienți. Capacitatea globală a pacientului (status cognitiv, mișcări funcționale) și mediul în care trăiește trebuie evaluate înainte de luarea unei decizii. 

FES poate reprezenta cheia independenței funcționale a pacienților neurologici care îi poate ajuta să se reintegreze în viașa socială, crescând încrederea în sine, în același timp, însă poate reprezenta o cauză de depresie, când așteptările pacienților depășesc posibilitățile reale.

# Meeting report: CAZ CLINIC DE NECROZĂ ASEPTICĂ DE CAP FEMURAL

**Authors:** Larisa Rarinca, Dan Alexandru Dumitrașcu, Delia Cinteză

**Affiliations:** * I.N.R.M.F.B. – Clinica Recuperare Medicală-Neurologie (IV)

Necroza avasculară reprezintă moartea celulelor osoase de la nivelul osului datorată întreruperii fluxului sanguin. Este caracterizată prin distrugerea structurilor osoase (a traveelor) osul fiind distrus, la acel nivel aparând durerea și pierderea funcției articulare. In momentul în care articulația se distruge (artc. CF este cel mai frecvent afectată) este necesară protezarea acesteia, osteonecroza nefiind o boala care se vindecă.

In cazul de față am examinat o pacientă în vârstă de 70 ani, sex feminin care s-a prezentat în clinică pentru durere la nivelul articulației coxo-femurale drepte cu iradiere inghinală și pe membrul inferior stâng până la nivelul genunchiului și dificultăți de ambulație și pentru care se încearcă realizarea unui diagnostic diferențial aldurerii cu caracter mecanic de la nivelul articulației coxofemurale sau periarticular, obiectivat prin examene paraclinice și semnele clinice ale pacientului și pentru stabilirea unei conduite terapeutice de recuperare adaptate cazului.

# Meeting report: MECANISMUL EXTENSOR AL GENUNCHIULUI – PATOLOGIE ȘI RECUPERARE

**Authors:** Andreea Romila, Laurentia Draghescu, Viorel Tutu-Pata, Rodica Scarlet, Dana Dimulescu

**Affiliations:** * I.N.R.M.F.B. – Clinica Recuperare Medicină Fizică și Balneologie (II)

Articulația genunchiului are o structură complexă, fiind alcatuită din mai multe compartimente, straturi musculare, tendoane, ligamente și structuri capsulare, motiv pentru care prezintă susceptibilitate mare la leziuni traumatice, afecțiuni legate de vârstă, artrite inflamatorii sau septice. Rolul principal al compartimentului anterior al articulației este cel de mencanism extensor, alcătuit în primul rând din tendonul cvadricipital, patelă și tendonul patelar.

Condromalacia patelară, sindromul de stres femuro-patelar, artroza femuro-patelară, tendinita rotuliană (Jumper’s knee), tendinita cvadricepsului, bursita patelară, subluxația acută recurentă sau cronică a rotulei, sindromul plicii sinoviale, maladia Osgood-Schlatter, sindromul Sinding-Larsen-Johansson, sindromul de tract iliotibial, toate pot reprezenta cauze ale durerii resimțită anterior la nivelul genunchiului, fiind necesar un diagnostic diferențial înainte de inițierea tratamentului de recuperare. Tratamentul chirurgical este rareori indicat, în 70% din cazuri existând o ameliorare evidentă a simptomatologiei în urma tratamentului conservator.

Având în vedere diversitatea substratului patologic, nu există un algoritm comun de tratament, deci un diagnostic corect reprezintă un punct de plecare bun pentru un tratament de recuperare corect aplicat și cu rezultate satisfăcătoare.

# Meeting report: BOALA CHARCOT-MARIE-TOOTH. CAZ CLINIC

**Authors:** Miruna Sandulescu, Delia Cinteză, Dan Alexandru Dumitrașcu

**Affiliations:** * I.N.R.M.F.B. – Clinica Recuperare Medicală-Neurologie (IV)

Boala Charcot-Marie-Tooth (CMT) este cea mai răspândită neuropatie periferică moștenită, cu o incidență de 17-40 la 100.000/persoane, fiind o boala genetic heterogenă, cu peste 40 de forme genetice, fiecare genotip având prezentare fenotipică variată. De regulă, transmiterea se face autozomal dominant, însa există și forme autozomal recesive și, foarte rar, mutații sporadice. 

A fost examinată o pacientă în vârstă de 47 de ani prezentând scădere de forță musculară la nivel distal atât la membrele superioare cât și inferioare, cu debut în copilărie și agravare progresivă. Diagnosticul de boala CMT a fost atribuit pe criterii clinice la vârstă de 8 ani, dar nu a fost niciodată confirmat genetic. Ca particularitate a cazului se evidențiază faptul ca nu a fost decelat caracterul ereditar al bolii, precum și faptul că prognosticul funcțional este bun, fiind însă grevat de o complianță relativa la tratament.

# Meeting report: STUDIU PRIVIND EFICIENȚA TERAPIEI CU UNDE DE ȘOC EXTRACORPOREALE ÎN MANAGEMENTUL SPASTICITĂȚII

**Authors:** Nica Sarah Adriana, Sîrghii Brîndușa, Meiu Lili, Ojoga Florina, Nartea Roxana

**Affiliations:** * I.N.R.M.F.B. – Clinica Recuperare Medicină Fizică și Balneologie (III)

Accidentul vascular cerebral şi urmările lui clinico-funcţionale sunt cele mai importante cauze ale dizabilităţii pe termen lung în Europa, fiind astfel o provocare pentru toţi medicii specialişti de recuperare. Clinica noastră tratează lunar 10-15 cazuri de pacienţi post accident vascular din faza post-acută până la cronică. Mai mult de jumătate dintre aceştia se confruntă cu o creştere a spasticităţii care le amplifică nivelul de dizabilitate.

Pentru a scădea spasticitatea şi a imbunătăţi starea clinico-funcţională am studiat efectul terapiei cu unde de şoc extracorporeale pe 12 pacienţi internaţi în clinica 3 a INRMFB în perioada 3 ianuarie – 9 martie 2017 care au suferit un AVC în ultimii 3 ani şi s-au internat pentru evaluarea şi tratamentul de specialitate al durerii, spasticităţii şi limitărilor din activităţile cotidiene. Am folosit scala Ashworth, VAS şi scorul ADL pentru evaluare înainte şi după tratamentul efectuat. Terapia a fost aplicată de două ori pe săptămână timp de 2 săptămâni la nivelul flexorilor palmari şi eminenţelor tenare şi hipotenare pentru membrul superior şi la nivelul joncţiunii musculotendinoase a muşchilor gastrocnemieni pentru membrul inferior.

Rezultatele pe termen scurt au arătat pentru majoritatea pacienţilor o îmbunătăţire a durerii, dar mai ales o scădere a spasticităţii urmate de o toleranţă mai bună a programului de kinetoterapie, fără modificări importante în scorul ADL. 

Limitele studiului sunt date de numărul scăzut de pacienţi incluşi, de lipsa evaluării efectului terapeutic pe termen mediu şi lung şi de absenţa unui studiu comparativ cu alte intervenţii cu scop similar.

# Meeting report: LEZIUNEA DE PLEX BRAHIAL-CORDON POSTERIOR (NERV RADIAL)

**Authors:** Lavinia Sodringa, Irina Petrușcă, Delia Cineză, Victorița Suciu, Sebastian Diaconescu, Stelian Sorica, Horia Lăzărescu

**Affiliations:** * I.N.R.M.F.B. – Clinica Recuperare Medicală-Neurologie (IV)

Introducere: Mecanismul de producere al traumatismelor de plex brahial este variat, cea mai frecventă cauza fiind tracțiunea acestuia, des întalnită în cazul accidentelor de circulație sau de muncă, mai rar prin rănirea directă prin plagă împușcată sau secțiuni prin plagă taiată. 

Obiective: Realizarea unui diagnostic diferențial al etiologiei leziunilor de plex brahial, obiectivat prin semnele clinice și examenele paraclinice ale pacientului, în vederea stabilirii unei conduite terapeutice adaptate cazului și a evaluării prognosticului de recuperare.

Material și metoda: Pacient în vârstă de 39 de ani, care a suferit in noiembrie 2016 un traumatism rutier soldat cu lezarea plexului brahial se prezinta in clinica noastra, in februarie 2017, cu deficit motor paretic de tip periferic la nivelul membrului superior drept.

Rezultate: Stabilirea diagnosticului etiologic diferențial al leziunii de plex brahial-cordon posterior (EMG,CT,Rx,Ecografie).

Concluzii: Necesitatea evaluării clinice amanunțite a pacientului și corelarea cu datele paraclinice în vederea stabilirii corecte a cauzei paraliziei de plex brahial-cordon posterior, este importantă în vederea realizării programului de recuperare dar și pentru stabilirea prognosticului funcțional.

# Meeting report: RECUPERAREA MEDICALĂ DUPĂ O FRACTURĂ PERTROHANTERIANĂ OPERATĂ LA UN PACIENT VÂRSTNIC

**Authors:** Stelian Sorica

**Affiliations:** * I.N.R.M.F.B. – Clinica Recuperare Medicală-Neurologie (IV)

Introducere: pacient vârstnic, 86 de ani, de sex masculin, care se prezintă în clinică pentru impotență funcțională la nivelul articulației șoldului drept, disfuncție de locomoție moderată post fractură pertrohanteriană dreaptă redusă ortopedic și chirurgical cu DHS.

Obiective: întocmirea unui plan de recuperare adaptat cu scopul de a preveni apariția complicațiilor, de a crește forța musculară/amplitudinea de mișcare la nivelul articulațiilor șoldurilor și a genunchiului drept; tonifierea mușchilor stabilizatori ai șoldului și genunchiului drept; creșterea toleranței la efort; îmbunătățirea capacității de ambulație, toate acestea crescând nivelul de indepentență a pacientului.

Materia si metoda: program complex de recuperare adaptat pacientului si patologiilor asociate (tratament medicamentos, educarea pacientului in ceea ce priveste amenajarea casei, kinetoterapie, ET, masaj) cu scopul de a creste nivelul functional al acestuia

Rezultate: Evoluție pe perioada internării a fost favorabilă cu îmbunătățirea forței musculare pe stabilizatorii șoldului și genunchiului și ameliorarea schemei de mers. Evoluția pe termen lung este în funcție de complianța pacientului la programul de recuperare și patologiilor asociate (FiA, stimulator cardiac).

Concluzii: tratamentul de recuperare trebuie să fie adaptat fiecărui pacient în parte având ca scop final ajungerea la un nivel cât mai înalt de independență.

# Meeting report: FRACTURA FEMURALĂ ATIPICĂ LA O PACIENTĂ CU OSTEOPOROZĂ POSTMENOPAUZĂ COMPLICATĂ TRATATĂ INCONSTANT

**Authors:** Elena Stănciulescu, Ana Maria Pavel, Viorel Petcu, Sebastian Diaconescu

**Affiliations:** * I.N.R.M.F.B. – Clinica Recuperare Medicală-Neurologie (IV)

Introducere: În timpul tratamentului cu bifosfonaţi au fost raportate fracturi atipice subtrohanteriene şi de diafiză femurală, în special la pacienţii care urmează un tratament de lungă durată pentru osteoporoză. Aceste fracturi transversale sau oblice scurte pot apărea oriunde de-a lungul femurului, imediat de sub trohanterul mic până imediat deasupra platoului supracondilar. Aceste fracturi apar în urma unui traumatism minor sau în absenţa unui traumatism, iar unii pacienţi prezintă durere la nivelul coapsei sau la nivel inghinal, asociată adesea cu aspecte imagistice de fracturi de stres, prezente cu săptămâni până la luni de zile înainte de apariţia unei fracturi femurale complete. Fracturile sunt adesea bilaterale; de aceea, la pacienţii trataţi cu bifosfonaţi la care s-a confirmat apariţia unei fracturi de diafiză femurală, trebuie examinat femurul contralateral. A fost raportată, de asemenea, vindecarea insuficientă a acestor fracturi.

Obiectiv: Exista o suspiciune de fractură femurală atipică la scurt timp dupa oprirea tratamentului cu durata de 3 ani cu Bonviva 150mg 1cp/lună la o pacientă cu istoric de osteoporoză postmenopauză și istoric de numeroase fracturi.

Material: Raportăm un caz al unei femei de 68 ani aflată la menopauză postanexectomie cu istoric de numeroase căderi de la propria înălțime, care se internează pentru durere având caracter mecanic la nivelul genunchiului și șoldului drept, dizabilitate funcțională la mers. APP semnificative: cancer col uterin, DZ tip 2 insulinonecesitant, HTA, sindrom mielodisplazic cu anemie secundară și istoric de numeroase fracturi prin căderi de la propria înălțime: 2003-fractură deschisă tibie și peroneu drept diafiză distală, 2009-fractură radius bilateral și 2016-fractură subtrohanterian (trohantero-diafizar) femur drept, fractură trohanteriană de femur drept și fractură de bazin în 2013 prin accident rutier. Este diagnosticată ulterior accidentului rutier, în 2013, cu osteoporoză (DXA: T score L1-L4=-3,3 si sold stg=-2,2) și primește tratament cu Bonviva 150mg 1cp/lună, alpha D3 0,5 mcg 1cp/zi, Ca lactic 500mg timp de 3 ani când DXA a indicat osteopenia.

Concluzii: Nu avem suficiente date imagistice și clinice legate de existența unor fracturi de stress, frecvent bilaterale apărute în urma unor traumatisme minore sau absența lor, pacienta neprezentând o accentuare a sindrom algic bilateral înainte de ultimul eveniment.

Trebuie luate în discuție alți factori de risc independent cum ar fi existent unor fracturi anterioare care duc la apariția instabilității funcționale. De asemenea, recomăndam monitorizarea markerilor biochimici ai turn-over osos (CTX urinar și osteocalcină plasmatică) fiind cunoscut faptul că se produce o revenire la valorile patologice ale ratelor de resorbție osoasă crescută, anterioare tratamentului, asociate cu osteoporoza din perioada de postmenopauză.

# Meeting report: MEDICINA FIZICĂ ȘI DE REABILITARE/REABILITARE MEDICALĂ/RECUPERARE, MEDICINĂ FIZICĂ ȘI BALNEOLOGIE – DE LA PERCEPȚIE LA SUCCES (REZIDENȚI, MEDICI, U.M.F.-URI, M.S., S.R.R.M., C.A.S.)

**Authors:** I.S. Stratulat

**Affiliations:** 52 Rehabilitation Department, Clinic Hospital C.F. Iași, România; 53 Faculty of Medicine, University of Medicine and Pharmacy “Grigore T. Popa” Iași, România; 54 Atmosphere Optics, Spectroscopy and Lasers Laboratory, Faculty of Physics, “Alexandru Ioan Cuza” University of Iași, România; 55 Center for Advanced Research in Bionanoconjugate and Biopolimers /Intel Centru/ Macromolecular Chemistry Institute „Petru Poni” Iași, România; 56 Emergency Military Hospital „Dr. Jacob Czihak” Iași, România/ Departament Rehabilitation.

Această specialitate medicală în cadrul căreia sunt abordate un număr important de patologii medicale post acute și cronice, cu sau fără evoluție dizabilitantă, necesită pentru tratamentul acestora un program de reabilitare modern și individualizat. Pentru ca acest plan terapeutic să corespundă cerințelor, este necesară existența unei echipe complexe (medic, asistentă, infirmieră, fiziokinetoterapeut, logoped, neuropsiholog, asistent social, asistent financiar, asistent îngrijire la domiciliu), echipă care are drept deziderat – cheie aplicarea corectă a principiilor de tratament individualizat fiecărui pacient, iar dizabilitatea să fie tratată cu costuri minime, de la actul medical până la societate. 

Interesul și percepția asupra propriei specializări a rezidenților din cadrul echipei de reabilitare medicală, a doctorilor din specialitate, a UMF-urilor, Ministerului Sănătății - Comisia de Specialitate cât și a societății profesionale S.R.R.M. - Societatea Română de Reabilitare Medicală a fost analizata prin percepția acestorara asupra stagiilor de pregătire teoretică și practică, durata acestora, bibliografia, legislația actuală și, nu în ultimul rând, factorii pe care îi consideră a fi de succes în specializare. Chestionare conținând anumite întrebări țintite pe aspecte cheie ale pregătirii în specialitate, pe condițiile actuale în care se desfășoară rezidențiatul, accesului la informație, a stagiilor de pregătire considerate a fi sau nu utile pregătirii ca rezidenți, și, nu în ultimul rând, pe inovațiile care ar trebui aplicate ca aceasta să corespundă dezideratelor de la teorie la practică și de la aceasta la pacienți. 

A fost analizată și existența surselor de finanțare pentru rezidenți, medici, C.A.S. și necesitatea unor restructurări. Factorul balneoclimatic, cu implicațiile în specialitate a fost analizat prin prisma resurselor de care beneficiază România în U.E. și în lume. 

# Meeting report: PHISICAL MEDICINE AND REHABILITATION/MEDICAL REHABILITATION/RECOVERY, PHYSICAL MEDICINE AND BALNEOLOGY – FROM PERCEPTION TO SUCCES (RESIDENTS, DOCTORS, MEDICAL UNIVERSITIES, MINISTRY OF HEALTH, S.R.R.M., C.A.S.)

**Authors:** I.S. Stratulat

**Affiliations:** 57 Rehabilitation Department, Clinic Hospital C.F. Iași, România; 58 Faculty of Medicine, University of Medicine and Pharmacy “Grigore T. Popa” Iași, România; 59 Atmosphere Optics, Spectroscopy and Lasers Laboratory, Faculty of Physics, “Alexandru Ioan Cuza” University of Iași, România; 60 Center for Advanced Research in Bionanoconjugate and Biopolimers /Intel Centru/ Macromolecular Chemistry Institute „Petru Poni” Iași, România; 61 Emergency Military Hospital „Dr. Jacob Czihak” Iași, România/ Departament Rehabilitation.

This medical specialty which addresses a number of acute and chronic medical conditions, with or without disabling evolution, requires for their treatment a modern and individualized rehabilitation program. In order for this treatment plan to meet the requirements, it is necessary to have a complex team (doctor, nurse, nurse, physiokinetotherapists, speech therapist, neuropsychologist, social worker, financial assistant, assistant nursing home), that has as essential goal the correct application of principles of individualized treatment for each patient, and to treat disability with minimal cost, from the medical act to society.

In this paper it was measured the interest and perception of residents over their own specialization within the medical rehabilitation team, doctors in the specialty from the universities, Ministry of Health - Specialised Commission and the professional society S.R.R.M. - Romanian Society of Medical Rehabilitation. Those perception was tested on theoretical and practical training sessions, their duration, bibliography, current legislation and, not least, the factors we consider to be successful for specialization. The project included a number of questionnaires containing specific questions targeted on key aspects of training in the specialty, the current conditions of the residency, access to information, the training sessions deemed to be or not useful for training as residents, and not last, the innovations that should be applied as it corresponds to the aspirations of theory to practice and to the patients.

It was analyzed also the existence of funding sources for residents, physicians, C.A.S. and the need for restructuration. The balneary factor with its implications was analyzed through specialized resources that Romania benefits from its location in the EU and the world.

# Meeting report: ANTRENAMENTUL MERSULUI CU AJUTORUL UNUI DISPOZITIV ROBOTIZAT

**Authors:** Victorița Marcu, Liliana Cioc, Delia Cinteză, Teona Olariu, Diana Manea, Bogdan Angheluță

**Affiliations:** * I.N.R.M.F.B. – Clinica Recuperare Medicală-Neurologie (IV)

Premise: Antrenamentul pe covor rulant este adesea utilizat în recuperarea mersului la pacienții cu afecțiuni neurologice plecând de la supoziția stimulării neuroplasticității prin influențarea activității centrului spinal generator de scheme de mișcare (spinal CPG) și prin folosirea principiului repetiției în consolidarea învățării și reînvățării. Totuși, acest tip de antrenament nu este pe deplin standardizat, indicațiile de aplicare nu sunt clare, iar unii cercetători pun la îndoială specificitatea orientată a acestui tip de antrenament.

Obiectivul acestui studiu este de a stabili indicațiile și eficacitatea antrenamentului cu ajutorul dispozitivului robotizat în reabilitarea mersului la pacienții cu afecțiuni neurologice.

Acest dispozitiv robotizat conține 4 module: “Gait Analysis” - modul pentru analiza mersului și pentru crearea de tipare de mers pentru pacienți; “Eye Gait” - modul pentru antrenarea activă a membrelor superioare în timpul ședintei de tratament; “Virtual Reality” - modul ce transpune pacientul într-un mediu familial; “Games” – interfață tip „jocuri” ce permite stimularea funcțiilor congitive și recuperarea într-un mod complex și unitar a funcțiilor de echilibru a pacienților.

Material și metoda: studiul, aflat încă în desfășurare, este unul de tip prospectiv. Pacienții incluși sunt pacienți cu afecțiuni neurologice, capabili să meargă cel puțin 5 pași cu sau fără sprijin, indiferent de gradul de spasticitate. Pacieții au fost evaluați din punct de vedere demografic, medical general, neurologic și funcțional. S-a procedat la înregistrări ale parametrilor mersului la începutul studiului și după 10 ședinte de antrenament cu ajutorul dispozitivului robotizat.

Rezultatele preliminare permit conturarea indicațiilor pentru acest tip de antrenament, a criteriilor pe care pacientul trebuie să le îndeplinească pentru ca antrenamentul să fie eficace. Studiul prezintă și metodologia standard aplicată, cu variații individuale privind gradul de suspendare și viteză de rulare a covorului.

Concluzii: antrenamentul mersului cu ajutorul dispozitivului robotizat, la pacienții cu afecțiuni neurologice, poate fi benefic dacă indicația de aplicare este adaptată nevoilor specifice ale pacienților. Mecanismul prin care acționează este cel de stimulare a neuroplasticității, dar rezultatul poate fi unul nedorit, dacă neuroplasticitatea nu este orientată spre atingerea unui scop funcțional adaptat fiecărui individ.

# Meeting report: PROGRAMUL DE REABILITARE ȘI CALITATEA VIEȚII LA PACIENȚII VÂRSTNICI CU GONARTROZĂ

**Authors:** Rodica Trăistaru, Otilia Rogoveanu, Diana Kamal, Constantin Kamal

**Affiliations:** * Universitatea de Medicină și Farmacie din Craiova

Obiective: Gonartroza reprezintă o problemă majoră socială și de sănătate și o cauză importantă de dizabilitate în rândul populației în curs de îmbătrânire, generând o povară financiară tot mai grea asupra sistemelor de sănătate în societatea modernă. In studiul nostru prospectiv, am evaluat starea clinică și funcțională și calitatea vieții, definită prin măsurarea performanței fizice și a parametrilor funcționali, înainte și după programul de reabilitare, la pacienții vârstnici cu gonartroză.

Material și metode: 66 de pacienți (42 femei, 24 bărbați), cu vârste cuprinse între 68 - 76 de ani au fost evaluați clinic, funcțional și imagistic (radiografie și ultrasonografie). Am folosit scala VAS pentru evaluarea durerii și indicele WOMAC pentru determinarea statusului funcțional. Performanța fizică a fost evaluată cu ajutorul testului de mers 6 minute (SMW) și Testul "Up & Go" cronometrat. Pacienții noștri au efectuat un program complex de reabilitare, 10 ședințe. Am luat în considerare toate comorbiditățile subiecților studiați. Pacienții au fost evaluați la momentul inițial (T1) la 2 (T2) și 12 (T3) săptămâni.

Rezultate: Parametrii studiați au avut o evoluție bună, mai ales în momentul T2. Analiza multivariată a arătat că valorile medii ale testului de mers 6 minute (SMW) și Testul "Up & Go" cronometrat s-au corelat statistic cu scorul scalei VAS și a indicelui WOMAC.

Concluzii: Rezultatele noastre confirmă efectul favorabil (clinic și funcțional) al programului complex de reabilitare efectuat la pacienții vârstnici cu gonartroză decompensată algic. Statusul funcțional s-a corelat cu performanța fizică. Ameliorarea statusului clinic și funcțional al genunchiului și aplicarea unui program terapeutic complex la pacienții vârstnici cu gonartroză, reprezintă două obiective majore pentru membrii echipei medicale, în vederea îmbunătățirii calității vieții la acești pacienți.

# Meeting report: REHABILITATION PROGRAM AND QUALITY OF LIFE IN ELDERLY KNEE OSTEOARTHRITIS PATIENTS

**Authors:** Rodica Trăistaru, Otilia Rogoveanu, Diana Kamal, Constantin Kamal

**Affiliations:** * University of Medicine and Pharmacy of Craiova

Objectives: Knee osteoarthritis is a major social and health problem and a cause of disability among the aging population, generating an increasingly heavy financial burden on health care systems in modern societies. In our prospective study, we assess the clinical and functional status and evaluate the quality of life defined by the physical performance measures and functional parameters, before and after rehabilitation program, in older patients with painful knee osteoarthritis (KOA).

Material and Methods: Sixty-six patients (42 women, 24 men), aged between 68 - 76 years, were evaluated clinical, functional and imagistic (X – rays and ultrasonography). We used VAS scale for pain and WOMAC index for functional status. The physical performance measures included the Six-Minute Walk Test (SMW) and the Timed "Up & Go" Test (TUG). Our patients performed a complex rehabilitation program, 10 sessions. We took into consideration all comorbidities of our studied subjects. Patients were evaluated at baseline (T1) and at 2 (T2) and 12 (T3) weeks. 

Results: The studied parameters had a good evolution, especially in T2 moment. Multivariate analysis showed that the mean values of Six-Minute Walk Test (SMW) and the Timed "Up & Go" Test (TUG) were correlated statistically with VAS score and WOMAC index.

Conclusions: Our results confirm the favorable complex effect (clinical and functional) of rehabilitation performed in the old patients with painful KOA. The functional status is correlated with physical performance. The clinical and functional status knee exam and the complex therapeutic program for all patients with KOA represent two major objectives of all medical team members for enhance the quality of life.

# Meeting report: RECUPERAREA MEDICALĂ LA UN PACIENT CU AVC HEMORAGIC ȘI TROMBOZĂ VENOASĂ PROFUNDĂ

**Authors:** Iulian Zaifu, Liliana Cioc

**Affiliations:** * I.N.R.M.F.B. – Clinica Recuperare Medicală-Neurologie (IV)

Introducere. Accidentul vascular cerebral reprezintă pierderea rapidă a funcției cerebrale datorită tulburărilor aportului de sânge la nivelul cerebral. Etiologia acestuia poate fi ischemică sau hemoragică, având ca urmare paralizia hemicorpului contralateral. 

Material și metodă. Pacientul D G, în vârstă de 54 de ani, de sex masculin, din mediul urban, consumator cronic de etanol. Pacientul se internează în clinica noastră pentru: deficit motor paretic al hemicorpului stang, deficit de locomoție și autoîngrijire moderat, durere cu caracter mecanic la nivelul genunchiului stâng. Pacientul este cunoscut în antecedente cu HTA neglijată terapeutic și suferă în data de 30.05.2016 un episod de pierdere a stării de conștiență, petrecut la volan, urmat de confuzie postcritică și deficit motor al hemicorpului stâng motiv pentru care se prezintă în UPU a Spitalului Universitar Elias unde primește îngrijiri medicale de specialitate și se realizează un CT ce decelează o acumulare hemoragică (60/40mm) asociind edem difuz perilezional.

In urma consultului NK, date fiind starea clinică bună și GCS=14-15, se decide urmărire și tratament medicamentos, însă la 5 ore de la consult starea pacientului se agravează GCS ajunge la 7 puncte motiv pentru care se solicită reevaluare NK și se repetă CT-ul ce evidențiează creșterea în volum a hematomului temporo-parietal (77mm fata de 60mm), edemul perilezional fiind în evoluție motiv pentru care se intervine chirurgical în vederea evacuării hematomului. Post chirurgical, CT-ul realizat în data de 3.06 evidențiează reducerea marcată a componentei hemoragice cu persistența unor mici acumulări hemoragice (25/15mm) pentru ca în data de 11.06 sa evidențieze dispariția acumulărilor hemorgice parenchimatoase temporo-parietale drepte și minimă deplasare a liniei mediene (aprox 5mm). Pe parcursul internării pacientul a efectuat termoterapie la nivelul cotului și pumnului, dimineața înainte de programul KT pentru a facilita mobilitatea articulară, masaj relaxant la nivelul membrului superior însoțit de elemente facilitatorii precum presiune pe tendonalele lungi și posturarea mâinii cu degetele în extensie și kinetoterapie.

Rezultate. Evoluția a fost favorabilă din punct de vedere funcțional rezultând îmbunatățirea transferurilor în pat, îmbunătățirea schemei de mers și a tulburărilor de echilibru. La nivelul membrului superior evoluția a fost lent favorabilă cu îmbunătățirea ușoară a abilităților.

Concluzii. Pacient cu AVC hemoragic cu un prognostic funcțional bun dar care asociează TVP pentru care primește tratament anticoagulant. Afecțiunile asociate ale pacientului influențează tratamentul, prognosticul funcțional și vital al pacientului.

# Meeting report: ARHITECTURA CA SUPORT PENTRU REABILITAREA COGNITIVĂ A PACIENŢILOR CU DEMENŢĂ

**Authors:** Mihaela ZAMFIR (Grigorescu), Mihai-Viorel ZAMFIR, Maria MOGLAN

**Affiliations:** * Departamentul Sinteza Proiectării de Arhitectură, Facultatea de Arhitectură, Universitatea de Arhitectură și Urbanism “Ion Mincu”; M.M.G.- Birou Individual de Arhitectură; e-mail: mmg_architecturestudio@yahoo.com; web: https://mihaelagrigorescu.wordpress.com/; ** Disciplina Fiziologie II-Neuroștiințe, Facultatea de Medicină, UMF “Carol Davila”; e-mail: mihai.zamfir@yahoo.com; *** Vicepreşedinte Societatea Română Alzheimer; e-mail: mariamoglan011@gmail.com

Demenţele constituie o problemă majoră de sănătate publică datorită prevalenţei ridicate şi impactului major asupra calităţii vieţii. Se estimează că între 5 şi 7% dintre persoanele vârstnice au o formă de demenţă ceea ce face ca medici din diverse specialităţi să vină în contact cu această patologie. Cele mai întâlnite forme de demenţă sunt demenţele Alzheimer secondate de demenţele vasculare. 

Pe lângă tratamentul farmacologic şi asistenţa de tip îngrijire, în ultima vreme se pune accent pe reabilitarea cognitivă a pacienţilor cu demenţă. Reabilitarea cognitivă este un program comprehensiv, orientat spre obiective, care include stimulare cognitivă, antrenament cognitiv şi alte abordări şi care îşi propune să îmbunătăţească funcţiile cognitive, memoria, atenţia şi concentrarea precum şi diferite abilităţi de viaţă cotidiană. Mai nou, în cadrul reabilitării cognitive, se pune accent pe rolul arhitecturii. Arhitectura şi designul de interior pot influenţa pozitiv procesul de reabilitare cognitivă alături de medicaţie şi consiliere psihologică, contribuind la creşterea calităţii vieţii persoanei vârstnice cu demenţă. 

Lucrarea investighează modul în care instrumente arhitecturale precum lumină, formă, culoare, textură, sunet pot contribui la realizarea unui mediu suportiv în reabilitarea cognitivă a vârstnicului cu demenţă în diferite cazuri: propria locuinţă, centre/clinici de reabilitare, cămine de tip nursing-home. Lucrarea deasemenea verifică în mod aplicat principii precum asigurarea unui spaţiu confortabil şi a unui mediu terapeutic, proiectarea optimă a căilor de circulaţie pentru evitarea dezorientării, reducerea agitaţiei şi dromomaniei, flexibilitate, accesibilitate, securitate şi siguranţă, estetică. 

În concluzie, cele 5 instrumente arhitecturale pot contribui la crearea unui mediu suportiv în reabilitarea cognitivă a pacientului cu demenţă atât ca acţiune individuală cât şi ca acţiune sinergică. O utilizare inteligentă a acestor instrumente îmbunătăţeşte orientarea temporo-spaţială, ameliorează simptomele depresive şi tulburările de somn care pot întârzia apariţia rezultatelor în reabilitarea cognitivă, diminuează agitaţia, cresc apetitul şi în final gradul de independenţă. Se remarcă o ierarhie a acestor instrumente, forma este mai recunoscută de pacienţii cu demenţă decât culoarea şi culoarea mai mult decât funcţiunea unui spaţiu. Fiecare tipologie de mediu, locuinţă, centru de reabilitare, cămin poate constitui subiect pentru studii particulare. 

Cuvinte cheie: arhitectură, instrument arhitectural, reabilitare cognitivă, demenţă, calitatea vieții.

# Meeting report: TRECUT, PREZENT SI PERSPECTIVE PRIVIND STANDARDIZAREA SI REGLEMENTAREA PROFESIEI DE FIZIOTERAPEUT IN CONTEXTUL RECUPERARII MEDICALE IN ROMANIA

**Authors:** Adriana Sarah NICA, Mariana CONSTANTINOVICI

**Affiliations:** * UMF Carol Davila – INRMFB, Bucuresti

Profesie in domeniul sanatatii recunoscuta la nivel international, fizioterapia este un important domeniu terapeutic sustinut in cadrul echipei si a programului de recuperare-reabilitare, in Romania, programele de formare profesionala a fizioterapeutilor desfasurandu-se, de-a lungul timpului, in sectiunile specifice in cadrul institutiilor de invatamant medical universitar si avand permanent la baza un curriculum specific, coerent si corespunzator modelului international. La nivel european si mondial exista diferite modele de reglementare profesionala, in functie de caracteristicile sistemelor de sanatate si de contextul legislativ, economic si cultural.

Pentru furnizarea unor servicii de sănătate cât mai performante și de calitate pentru pacienții cu disfuncții la nivel locomotor din domeniul reabilitării – recuperării, medicinei fizice si balneologiei, in clinici de recuperare cu paturi, în regim ambulator sau în cadrul programelor de recuperare la domiciliu si pentru reducerea variabilității de practică profesională medicală între profesioniștii din domeniul fizioterapiei (fizioterapeuți și asistenți de fizioterapie) se justifica nevoia de standardizare si reglementare a activității profesionale în domeniu, precum si dezvoltarea si implementarea unor protocoale specifice sau a unor ghiduri de buna practica in domeniul terapiei fizicale si al recuperarii.

In acest context, incercam sa prezentam cateva modele si posibilitatile juridice justificate pentru practica de fizioterapie, adaptat specificului sistemului de sanatate din Romania.

# Meeting report: PAST, PRESENT AND PERSPECTIVES ON STANDARDIZATION AND REGULATION OF PHYSIOTHERAPY PROFESSION IN THE CONTEXT OF PHYSICAL MEDICINE AND REHABILITATION IN ROMANIA

**Authors:** Adriana Sarah NICA, Mariana CONSTANTINOVICI

**Affiliations:** * UMF Carol Davila – INRMFB, Bucuresti

Profession in healthcare domain internationally recognized, physiotherapy is an important therapeutic area supported within the rehabilitation program in the rehabilitation team, in Romania, training programs of physiotherapists unfolding, over time, in specific sections in university medical education institutions and permanently based on specific curriculum, consistent and appropriate to the international model. There are different models of professional regulation at European and international level, depending on the characteristics of health systems and legislative, economic and cultural context.

The need for the provision of performant, qualitative and safety health services for patients with locomotor dysfunction in rehabilitation, inpatient, outpatient basis or within rehabilitation programs at home and for reducing variability professional practice care between professionals physiotherapy (physiotherapists and physiotherapy assistants) justify the imnpportance and the need for standardization and regulation of professional activity in the field. Also, the development and implementation of specific protocols or guidelines on best practice in the field of physical therapy and rehabilitation.

In this context, we try to present some models and reasoned legal possibilities for the practice of physiotherapy, adjusted to the health system in Romania.

# Meeting report: TERAPIA FIZICALA IN RECUPERAREA CRPS I DE MEMBRU SUPERIOR

**Authors:** Mariana CONSTANTINOVICI, Brindusa MITOIU, Roxana NARTEA, Gilda MOLOGHIANU, AS NICA

**Affiliations:** * UMF Carol Davila – INRMFB, Bucuresti

Sindromul dureros regional complex tip I de membru superior, dezvoltat mai ales posttraumatic, cu implicarea sistemului nervos, senzorial, motor si autonom, determina frecvent probleme disfunctionale la nivelul mainii, ce pot avea un impact functional important asupra abilitatii gestuale cotidiene, asociind durere si dizabilitate, prin limitarea activitatilor si participarii. Terapia fizicala este o componenta importanta in managementul de recuperare al sindromului dureros regional complex si se bazeaza pe programul de mobilizare si diferite modalitati terapeutice de tip nonfarmacologic, fizical, avand ca obiectiv ameliorarea manifestarilor algice si prevenirea problemelor fizice secundare determinate de limitarea de mobilitate. Vor fi prezentate date actuale din literatura privind evaluarea functionala, modalitatile specifice de terapie fizicala si recomandarile ghidurilor de la nivel international si rezultatele terapeutice ale programelor de recuperare pentru pacientii cu sindrom dureros regional complex.

# Meeting report: PHYSICAL THERAPY IN UPPER LIMB CRPS I REHABILITATION

**Authors:** Mariana CONSTANTINOVICI, Brindusa MITOIU, Roxana NARTEA, Gilda MOLOGHIANU, AS NICA

**Affiliations:** * UMF Carol Davila – INRMFB, Bucuresti

Complex Regional Pain Syndrome type I in the upper limb develops especially after trauma and involves sensory, motor and autonomic components of the nervous system. It often causes hand dysfunction and may have a functional impact in the area of daily gestures and abilities, associating pain, activity limitations and participation restriction - disability. Physical therapy is an important player in the management of complex regional pain syndrome. It consists of mobilisation programs and various specific non-pharmacological therapeutic physical modalities, aiming pain relief and the prevention of secondary physical dysfunction caused by upper limb disuse. There will be presented current data from international medical literature regarding functional evaluation, specific physical therapy methods and recommendations of international guidelines and treatment outcomes of rehabilitation programs for patients with complex regional pain syndrome.
